# Development of a bispecific antibody–drug conjugate targeting EpCAM and CLDN3 for the treatment of multiple solid tumors

**DOI:** 10.1186/s40164-025-00624-9

**Published:** 2025-03-08

**Authors:** Meiying Luo, Xiaohuan Wang, Guoji Yu, Jing Ji, Long Li, Fan Song

**Affiliations:** 1 State Key Laboratory of Nonlinear Mechanics, Beijing Key Laboratory of Engineered Construction and Mechanobiology, Institute of Mechanics, Chinese Academy of Sciences, No. 15, North Fourth Ring Road West, Beijing, 100190 China; 2 Department of Rehabilitation Medicine, Peking University Third Hospital, 49 North Garden Road, Beijing, 100191 China; 3 Inner Mongolia Metal Material Research Institute, Yantai, 264003 Shandong China; 4 School of Biological Science and Medical Engineering, Beihang University, Beijing, 100191 China; 5 Guangzhou Institute of Cancer Research, the Afiliated Cancer Hospital, Guangzhou Medical University, Guangzhou, 510095 China; 6 Center of Materials Science and Optoelectronics Engineering, School of Engineering Science, University of Chinese Academy of Sciences, Beijing, 100049 China

**Keywords:** EpCAM, CLDN3, Antibody–drug conjugate, Dxd, Solid tumor

## Abstract

Epithelial cell adhesion molecule (EpCAM), a tumor antigen for antibody–drug conjugates (ADCs), is highly expressed in many epithelial cancers. However, the clinical progress of EpCAM ADCs has been challenging, primarily due to their toxicity in normal high-expression tissues such as the gastrointestinal tract. CLDN3 is highly co-expressed with EpCAM in various human malignancies, coupled with its minimal presence in normal tissues, rendering it an ideal target for developing potent therapeutic ADCs. Here, we developed a bispecific ADC (BsADC) targeting EpCAM and CLDN3, designed to avoid toxicity in normal tissues with high EpCAM expression. The parental monoclonal antibodies (mAbs) were screened for high binding and endocytosis activities on tumor cell lines. We then modified them into monovalent structures and selected clones with decreased binding and endocytosis activities. We combined these clones into bispecific antibodies (BsAbs) and finally chose the molecules with restored binding and endocytosis activities as lead molecules. The BsADCs were generated by conjugating the Drutecan (Dxd) to BsAbs via a cleavable linker. These conjugates exhibit potent binding and effectively inhibit the growth of tumor cells with high levels of both EpCAM and CLDN3, indicating their anti-tumor efficacy. Importantly, they show weak binding to cells with high EpCAM but low CLDN3, implying minimal toxicity to normal tissues with elevated EpCAM expression. Moreover, the BsADCs displayed advantageous pharmacokinetics and low toxicity in mice. These findings position the BsADCs targeting EpCAM and CLDN3 as promising candidates for treating multiple solid tumors.

**Supplementary Information:**

The online version contains supplementary material available at 10.1186/s40164-025-00624-9.

## Introduction

Antibody–drug conjugates (ADCs) represent a significant advancement in targeted cancer therapy, combining the specificity of antibodies with the potency of cytotoxic drugs [1, 2]. In recent years, ADCs have become powerful tools for tumor treatment due to their ability to selectively deliver cytotoxic agents directly into cancer cells while preserving healthy tissues [3, 4]. However, traditional ADCs that target receptors expressed on both tumor cells and certain non-malignant tissues (such as EGFR and TROP2) are often associated with unavoidable on-target off-tumor toxicities, leading to dose reductions or treatment discontinuation [5, 6]. Bispecific antibodies (BsAbs) recognize two epitopes or antigens [7, 8]. Conjugating payloads on BsAbs to yield bispecific ADCs (BsADCs) with improved specificity and/or internalization is a new research area expected to overcome existing limitations, such as endocytosis, toxicity, and drug resistance to ADCs [9, 10]. Simultaneous targeting of two different antigens using a BsADC could offer multiple advantages. When appropriate targets are chosen, BsADCs might be capable of more tumor-specific binding owing to limited expression of both target antigens by non-malignant cells and/or promoted payload uptake, thereby minimizing the risk of toxicities in non-malignant tissues [11]. Furthermore, engaging multiple antigens could elicit a synergistic effect that might not be feasible by targeting either antigen individually [12, 13].

Epithelial cell adhesion molecule (EpCAM), a homophilic cell–cell adhesion glycoprotein, is a well-known tumor antigen expressed in various types of epithelial tumors [14, 15]. Its elevated levels on the surface of cancer cells are associated with increased cell proliferation, reduced cell adhesion, and promotion of tumor growth and metastasis [16–18]. It is important to note that EpCAM is also expressed, albeit at lower levels, in certain normal tissues, which makes EpCAM a compelling but historically “difficult-to-drug” target [19]. Many techniques were applied to alleviate the on-target off-tumor toxicities associated with targeting EpCAM. Probody drug conjugates (PDCs) are masked, protease-activatable antibody prodrugs designed to localize drug activity to the tumor microenvironment and minimize interaction with healthy tissues [20, 21]. The potential of developing BsADCs to reduce binding in normal tissues and enhance specific binding to tumor tissues has not yet been explored.

Claudin3 (CLDN3) is a member of the membrane protein claudin (CLDN) family, which comprises 27 unique proteins characterized by four transmembrane domains and two extracellular loops [22]. Various members of the CLDN family are located at tight junctions between epithelial cells, where they are believed to play an essential role in barrier functions [23]. More recently, CLDN18.2 and CLDN6 are recognized as promising tumor-associated antigen (TAA) genes, serving as potential targets for cancer therapy [24–26]. Correspondingly, numerous ADC products targeting these two genes are under development, with several in the late stages of clinical trials [25, 27]. CLDN3 is highly expressed in multiple human cancers such as ovarian, breast, prostate, and pancreatic cancers [28–30]. It contributes to tumorigenic potential by promoting cell proliferation, migration, and invasion while inhibiting apoptosis [31, 32]. However, its expression level in normal tissues is relatively low. This differential pattern demonstrating higher expression levels within malignant versus normal adult tissue makes CLDN3 an attractive target for developing antibody-based therapeutics, such as ADCs that can selectively deliver cytotoxic agents directly into these overexpressing cells, thereby providing more effective treatment options.

Here, we generated a BsADC targeting EpCAM and CLDN3 to deliver potent toxin payload more precisely to the tumors and mitigate on-target off-tumor toxicity. This novel BsADC demonstrated significant cytotoxicity against various tumor cell lines expressing EpCAM and CLDN3 both in vitro and in vivo. Notably, compared to the EpCAM-ADC, this BsADC also showed good safety in the toxicology study in mice. Furthermore, this BsADC exhibited favorable pharmacokinetic properties, making it a promising candidate for future treatment of patients with tumors expressing EpCAM and CLDN3.

## Materials and methods

### Cell lines and culture conditions

The human HEK293T (CRL-3216), SNU-5 (CRL-5973), OVCAR-3 (HTB-161), SK-OV-3 (HTB-77), MCF7 (HTB-22), MDA-MB-468 (HTB-132), MDA-MB-231 (HTB-26), COLO-201 (CCL-224), HT-29 (HTB-38), HCT116 (CCL-247), H1781 (CRL-5894), A549 (CCL-185), Capan-1 (HTB-79), Panc-1 (CRL-1469), PC-3 (CRL-1435), and DU-145 (HTB-81) cell lines were obtained from the American Type Culture Collection (ATCC) (Manassas, VA, USA). NUGC-4 (JCRB0834) was obtained from the Japanese Collection of Research Bioresources cell bank (Tokyo, Japan). All cells were cultured in DMEM or RPMI-1640 medium containing 10% fetal bovine serum (FBS; Invitrogen), 1% penicillin–streptomycin (Gibco-BRL) and 2 mM l-glutamine at 37 °C with 5% CO_2_. Following the manufacturer's instructions, Transfections were performed using the Lipofectamine 3000 transfection kit (Thermo Fisher) according to the manufacturer's instructions. All cells were confirmed to be free of mycoplasma contamination by using a cell culture contamination detection kit (Thermo). None of the cell lines used in this study was found in the database of commonly misidentified cell lines maintained by ICLAC and NCBI Biosample.

### Generation of EpCAM and CLDN3 monoclonal antibody

To generate the anti-EpCAM and anti-CLDN3 monoclonal antibody, EpCAM or CLDN3 knockout C57BL/6J mice were immunized with human EpCAM or CLDN3 mRNA lipid nanoparticles (LNPs) every two weeks, using AddaVax (InvivoGen) as an adjuvant [33]. After three times immunization, splenocytes were isolated from high-titer mice and fused with the myeloma cell line. Positive hybridomas were obtained by detecting binding to HEK293T-EpCAM or HEK293T-CLDN3 overexpressing cells. Then, RNAs were extracted using the RNeasy mini kit (Qiagen), and the corresponding cDNAs were generated using high-capacity cDNA reverse transcription kits (Thermo Fisher). The unique mouse monoclonal antibodies binding to human EpCAM or CLDN3 were identified after sequencing. The anti-EpCAM or anti-CLDN3 antibody was humanized into a full-size human IgG1 and produced by CHO-S cells.

### Generation of EpCAM X CLDN3 BsAbs

The BsAbs were generated in-house using the Knob-into-hole and the CrossMab techniques [34]. The constructs were transfected into the CHO-S cells according to the published transient transfection procedure [35]. One week posttransfection, the supernatant of the culture was harvested for processing while the cell viability dropped to ~ 50%, according to the literature [36]. Expression was analyzed by SDS-PAGE as needed. The BsAbs were purified by a Protein A Plus Agarose (Thermo Fisher).

### Generation and characterization analysis of EpCAM X CLDN3 BsADC

EpCAM X CLDN3 BsADCs were synthesized by conjugating the DXd payload with the BsAbs. The control IgG-ADC was synthesized using a human IgG isotype control monoclonal antibody. To ensure comparability of DAR values between these two ADCs, the IgG-ADC used the same drug linker (GGFG) as BsADCs. Derivatizations of the side chains of cysteine residues accomplished the bioconjugation. Partial reduction of these disulfide linkages provides a distribution of free thiols that can be functionalized with the maleimide handle on the linker. The ADC was further characterized by hydrophobic interaction chromatography.

### Plasmids and generation of overexpression cells

The full-length cDNA sequences of hEpCAM, hTrop2, cynoEpCAM, hCLDN3, hCLDN4, hCLDN6, hCLDN9, and cynoCLDN3 were constructed into the pCDH-CMV-MCS-EF1-Puro vector. The expression vectors were transduced into HEK293T cells and selected for survival in a medium containing puromycin (1 μg/mL). The positive cells were sub-cloned to obtain a stable and uniform colony of overexpressed cells.

### RNA extraction, cDNA synthesis, and real-time PCR analysis

Total RNAs from cultured cells were isolated with the RNeasy Mini Kit (Qiagen 74104), and 1 µg of total RNA was used for cDNA synthesis using a reverse transcription kit (Promega) according to the manufacturer's instructions. Quantitative real-time PCRs were conducted using iQ SYBR Green Master Mix (Bio-Rad). Samples were obtained and analyzed on the CFX96 Touch Real-Time PCR Detection System. The gene expression levels were normalized to actin. The primer sequences used for PCR were: EpCAM forward, 5′-AAACTTGGGAGAAGAGCAAAACC-3′ and EpCAM reverse, 5′-CAGTTGATAACGCGTTGTGATC-3′; CLDN3 forward, 5′-TTGCCCATGTGGCGCGTGT-3′ and CLDN3 reverse, 5′-AAGGAACAGCACGCCTGCC-3′; actin forward, 5′-GCTCGTCGTCGACAACGGCT-3′ and actin reverse, 5′-CAAACATGATCTGGCTCATCTTCTC-3′.

### Cytotoxic assay in vitro

Cell viability was evaluated using a CCK-8 kit (Sigma, 96992). The OVCAR-3, NCI-H1781, A549, HCT116, and SNU-5 cell lines were seeded into 96-well plates and incubated overnight at 37 ℃. After 24 h, the cells were exposed to the antibodies or ADCs for 72 h. Then, the cytotoxicity of antibodies and ADCs was determined by quantifying cell viability with the CCK-8 kit according to the manufacturer's protocol. Absorbance readings at 450 nm were measured using a microplate reader. The percentage of cell viability was calculated using the following formula: cell viability (%) = [(sample group absorbance−blank group absorbance)/(control group absorbance−blank group absorbance)] × 100%.

### Cell surface binding measured by FACS

To test the binding capacity of the antibody to tumor cells, the antibody was diluted from an initial concentration of 5 μg/mL, and a series of gradient dilutions were performed. After incubation with the tumor cells, the antibody fluorescence signal was measured using BD flow cytometry, and the collected data was analyzed using Flow-JO software to evaluate the binding capabilities.

### Internalization assay

The pHAb reactive dyes were used for monitoring receptor-mediated antibody endocytosis (Promega, G9841). Briefly, antibodies were conjugated with the pHAb reactive dyes according to the manufacturer's protocol. The concentration of antibodies was adjusted to 5 μg/mL and then incubated with tumor cells. Upon receptor-mediated internalization, antibody-pHAb Dye conjugates traffic to the endosome and lysosome where pH is acidic, causing the pHAb Dye to fluoresce. The fluorescence was detected by the flow cytometry.

### Affinity assay

The surface plasmon resonance (SPR) technique was employed using a BIAcore® 8 K instrument (manufactured by BIAcore, Uppsala, Sweden) to evaluate the affinity of the direct interaction between EpCAM and CLDN3 candidate antibodies and antigens. The antibodies to be tested were diluted to 3 μg/mL; the antigens were diluted to 200 nM and then serially diluted. The final concentrations were 200 nM, 100 nM, 50 nM, 25 nM, 12.5 nM, 6.25 nM, 3.125 nM and 1.5625 nM. The antibodies were immobilized on a BIAcore CM5 sensor chip through the standard amine coupling method. Subsequently, the response signal of the reference flow cell was subtracted, and data analysis was performed using BIAevaluation 3.2 software.

### ADCC assay

ADCC activity was evaluated using the LDH release assay. NK cells isolated from human PBMC (Ori Biotech, FPBO03F-C-INVIVO, donor ID Z0713) using a cell isolation kit (Thermo, 11349D) were used as effector cells, and OVCAR-3 and HCT116 cells were used as target cells. The effector and target cells were mixed according to the number of cells 10:1, and different antibody concentrations were added for co-incubation. After being cultured at 37 °C and 5% CO_2_ for 18 h, the cell supernatant was collected for LDH detection (Thermo, C20300), and the ADCC effect of the antibody was analyzed. The percentage of cell viability was calculated using the following formula: cell viability (%) = [(sample group absorbance−blank group absorbance)/(control group absorbance−blank group absorbance)] × 100%.

### ADCP assay

ADCP activity was evaluated using the ADCP reporter bioassay (THP-1) kit (Promega). THP-1 cells were used as effector cells, and OVCAR-3 and HCT116 cells were used as target cells. The OVCAR-3 and HCT116 cell lines were seeded into a 96-well plate, and the diluted antibodies and THP-1 cells were transferred into the plates and mixed well by pipetting. Cover the assay plates with lids and incubate at 37 °C, 5% CO_2_ for 4 h. After incubation, the ADCP activity is compatible with Bio-Glo-NL™ Luciferase Assay Reagent and a microplate reader. The percentage of cell viability was calculated using the following formula: cell viability (%) = [(sample group absorbance−blank group absorbance)/(control group absorbance−blank group absorbance)] × 100%.

### CDC assay

The OVCAR-3 and HCT116 cell lines were seeded into a 96-well plate and incubated overnight. Then 25 μL of the diluted antibodies were added, and the plates were incubated for 1 h at 4 °C. Human complement was diluted and added at 25 μL per well, and the plates were incubated for 1 h at 37 °C in the CO_2_ incubator. After incubation, the cellular ATP level was measured using CellTiter-Glo Reagent and a microplate reader. The percentage of cell viability was calculated using the following formula: cell viability (%) = [(sample group absorbance−blank group absorbance)/(control group absorbance−blank group absorbance)] × 100%.

### Growth inhibition of tumor xenografts in vivo

NU/NU Nude and C57BL/6J mice were acquired from Vital River Laboratories (Beijing). All animal studies followed institutional guidelines of the Animal Care and Use Committee (IACUC) of the Institute of Mechanics, Chinese Academy of Sciences, and Peking University Third Hospital. The related tumor cells (2 × 10^6^ cells) were implanted subcutaneously into immunodeficient mice (7 weeks old). Once tumor size reached 150 mm^3^, the tumor-bearing mice were randomized to achieve equal average tumor size and variance in the treatment group. ADCs were intravenously administered twice at a 7-day interval. Tumor volume (1/2 × length × width^2^) was monitored with caliper measurement twice weekly.

### Pharmacokinetics (PK) analysis

To evaluate the stability of ADC in vivo, a single dose of BsADC and control ADC were injected into female BALB/c mice. The total antibody concentration of serum was detected at specified time points. The concentrations of the total antibody in plasma were determined with a validated ligand binding assay, and the concentrations of DXd in plasma were determined with a validated liquid chromatography-tandem mass spectrometry (LC–MS/MS) method.

### Toxicological analysis in vivo

The toxicity of BsADC was evaluated in the mice model. 5-week-old female nude mice were injected with vehicle control (PBS) or ADC (6 mg/kg on days 0, 4, and 7). Five mice from each group were collected for toxicology studies one week after ADC injection. Body weight measurements were taken for a cohort of five mice per group, with weight changes monitored from the initial day of injection through day 21. Additionally, another set of five mice per group was euthanized three days after the final injection to evaluate serum levels of alanine transaminase (ALT) and aspartate transaminase (AST). Subsequently, the heart, liver, spleen, lungs, and kidneys were carefully excised, rinsed with PBS two to three times to remove debris, and then fixed in a 4% paraformaldehyde solution. The organs were maintained at room temperature before undergoing hematoxylin and eosin (H&E) staining for histological examination.

### Statistical analysis

Statistical analyses were conducted using GraphPad Prism software (version 8). Each cell line experiment was independently repeated more than three times, with each condition tested in technical triplicate. Data are presented as means ± SD. Differences between groups were assessed using ANOVA with Tukey's post-hoc test. *p*-value less than 0.05 was considered statistically significant.

## Results

### EpCAM and CLDN3 are co-overexpressed in various types of tumors

**Fig. 1:**
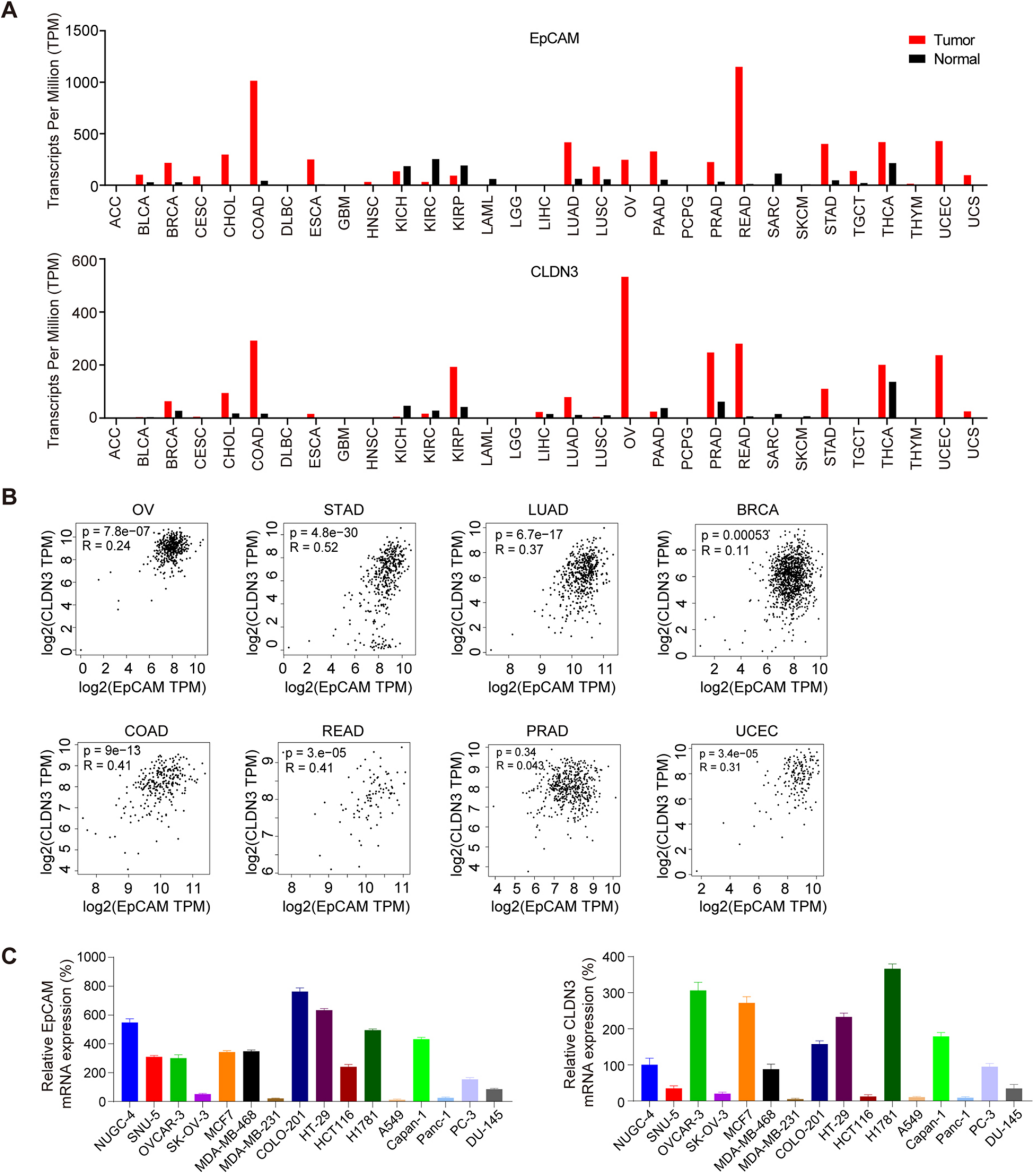
EpCAM and CLDN3 are co-expressed at high levels in various types of tumors. **A** Expression of RNA transcription of EpCAM and CLDN3 in the GEPIA cancer tissue sample database. **B** EpCAM and CLDN3 are co-expressed at high levels in different tumors. The correlation analysis results also indicate that EpCAM and CLDN3 are highly expressed concurrently in the tumors of the majority of patients. **C** Relative RNA expression of EpCAM and CLDN3 in different tumor cell lines. RNA from 16 tumor cells was isolated, and the expression of EpCAM and CLDN3 was detected by RT-PCR. Abbreviations for different cancers are shown in supplementary Table 1

EpCAM exhibits widespread and elevated expression across a spectrum of cancers, with a particular emphasis on colorectal malignancies, as evidenced by data from the Gene Expression Profiling and Interactive Analyses (GEPIA) database (Fig. 1A). EpCAM is also moderately expressed in normal tissues, predominantly in the gastrointestinal tract, kidneys, and pancreas. The bispecific EpCAM X CD3 antibody, Solitomab, has been associated with gastrointestinal toxicities in a Phase I clinical trial, leading to the discontinuation of the study. This adverse outcome is likely attributed to the presence of EpCAM in the normal gastrointestinal tissue. To address this issue, a comprehensive analysis was conducted to identify suitable tumor-associated targets for developing BsADC. This approach combines the targeting capabilities of two antigens, thereby enhancing tumor-specific binding affinity and significantly reducing the potential for toxicity in normal tissues. In the spectrum of 33 human cancer types, CLDN3 demonstrates an expression pattern that closely mirrors EpCAM’s (Fig. 1A).

We further analyzed the expression of EpCAM and CLDN3 in several cancers, including ovarian, gastric, and colorectal cancers. As shown in Fig. 1B and Figure S1, both EpCAM and CLDN3 are highly expressed in these tumors. Meanwhile, we also detected the expression of EpCAM and CLDN3 in 16 tumor cell lines by RT-PCR (Fig. 1C). We found that EpCAM and CLDN3 have a pronounced co-expression in various tumor cells, such as OVCAR-3 ovarian cancer cells and NCI-H1781 lung cancer cells. Conversely, we also observed a low co-expression in other cell lines, such as SK-OV-3 ovarian cancer cells and A549 lung cancer cells (Fig. 1C). We further detected the protein expression levels of EPCAM and CLDN3 on these tumor cells by FACS, and the results were consistent with mRNA expression (Fig. S2). These results demonstrated a strong correlation in the expression levels of EpCAM and CLDN3 across tumor cells. Notably, among all the tested cells, the difference between EpCAM and CLDN3 was found in HCT116 cells, with high expression of EpCAM and low expression of CLDN3. Taken together, these results suggest that CLDN3 is a promising TAA that can be targeted to develop BsADC with EpCAM to treat multiple solid tumors.

### Parental antibody selection and procedure for generating BsADCs

In order to construct the EpCAM X CLDN3 BsAb, we first obtained the parental mAbs of EpCAM and CLDN3 (Fig. S3). EpCAM is a single transmembrane protein with only one homologous gene Trop2. The monoclonal antibodies against EpCAM were prepared by immunizing EpCAM knockout C57BL/6J mice to generate antibodies cross-react with both human, cynomolgus monkeys, and mouse EpCAM, and 31 antibodies with high binding ability to human and cynomolgus monkeys EpCAM were obtained. To test the toxicity of ADCs in mouse models, these selected antibodies also cross-reacted with mouse EpCAM. As shown in Figure S4A, one representative EpCAM mAb binds to HEK293T cell lines overexpressing human, cynomolgus monkey and mouse EpCAM but not to wild-type HEK293T cells. Additionally, the mAb also does not bind to TROP2-overexpressed cell lines (Fig. S4A), suggesting that EpCAM mAb can specifically recognize EpCAM. We further evaluated the binding capability of EpCAM mAb to different human cancer cells. As shown in Figure S4B, EpCAM mAb binds strongly to OVCAR-3 cells and NCI-H1781 cells with high EpCAM expression but weakly to A549 cells with low EpCAM expression, thereby reaffirming its specificity for EpCAM. Given that antibody–drug conjugates (ADCs) rely on the internalization of antibodies via endocytosis to deliver their payloads into tumor cells, we proceeded to assess the endocytic activity of the antibody. Consistent with its binding profile, the EpCAM mAb displayed pronounced internalization in OVCAR-3 and NCI-H1781 cells, in contrast to the lower endocytic activity observed in A549 cells (Fig. S4C).

The CLDN3 protein, composed of 220 amino acids, features two extracellular domain (ECD) loops, as depicted in Figure S5A. Alignment with other family members reveals that ECD1 exhibits higher homology than ECD2, and CLDN3 shares 90% homology with CLDN4 and 80% homology with CLDN6 and CLDN9 in the ECD1 region (Fig. S5B). Consequently, the development of antibodies with high specificity for CLDN3 is of paramount importance. Anti-CLDN3 monoclonal antibodies were generated by immunizing mice with human CLDN3 mRNA lipid nanoparticles (LNPs), and 25 antibodies specifically binding to both human, cynomolgus monkey and mouse CLDN3 were obtained. These CLDN3-mAbs also did not bind to CLDN4, CLDN6, and CLDN9 (Fig. S5C), demonstrating that CLDN3-mAb can specifically recognize CLDN3. Next, we analyzed the binding ability of CLDN3-mAb to different human cancer cells. As shown in Figure S5D, CLDN3-mAb strongly binds to high CLDN3-expressing OVCAR-3 cells and NCI-H1781 cells, while relatively weak binds to low CLDN3-expressing A549 cells. Similarly, CLDN3-mAb showed high internalization activities in OVCAR-3 and NCI-H1781 cells, and relatively low endocytosis activities in A549 (Fig. S5E).

**Fig. 2:**
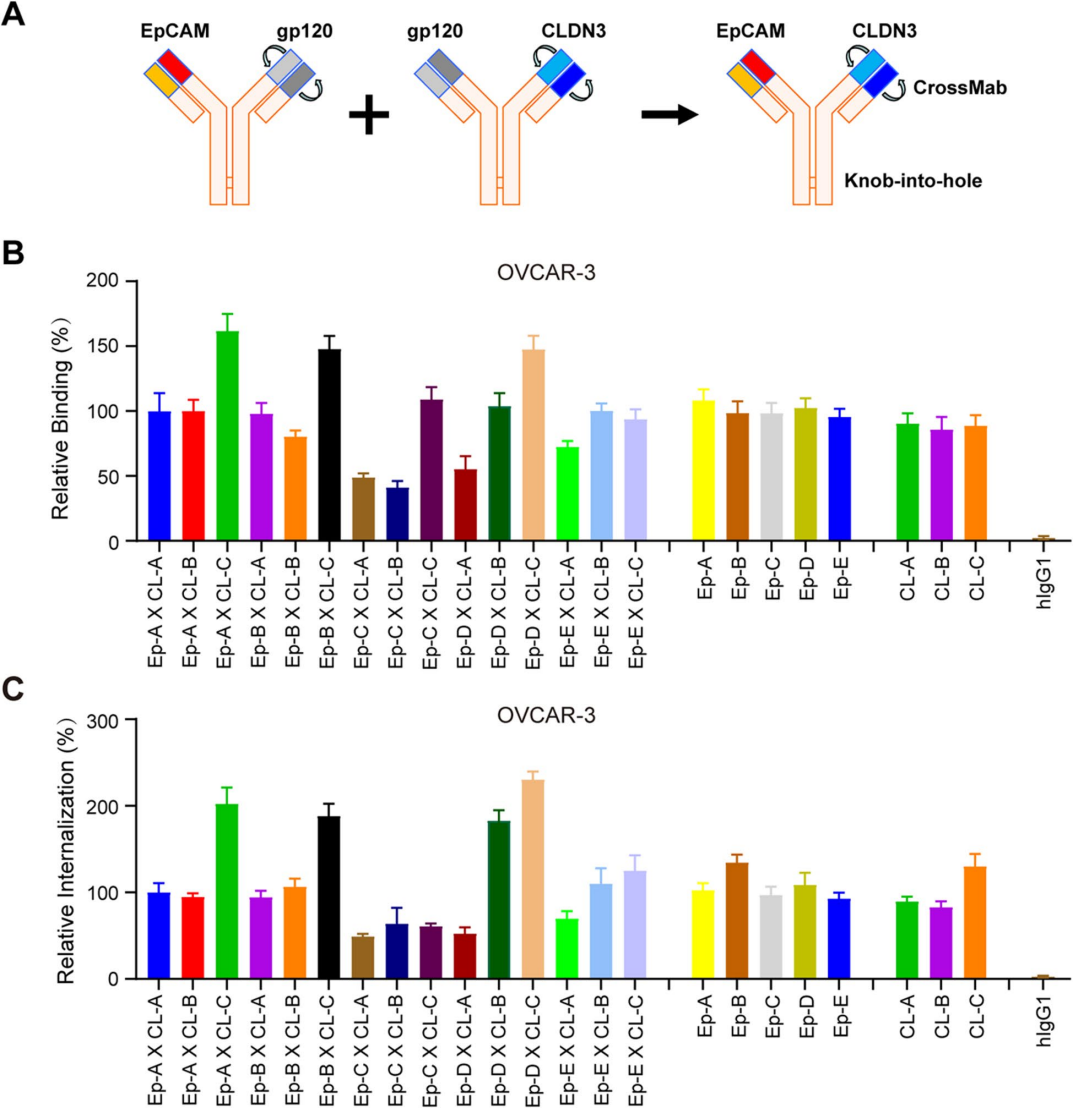
Development and Characterization of EpCAM X CLDN3 BsAbs. **A** Structure diagram of EpCAM X CLDN3 BsAb. Knob-into-hole and crossmab technologies are used respectively to prevent mismatching of heavy and light chains. **B** The binding activities of EpCAM X CLDN3 BsAbs were analyzed in OVCAR-3 cell lines at a concentration of 5 μg/mL. The EpCAM and CLDN3 monoclonal antibodies were used as controls. **C** The endocytic activities of EpCAM X CLDN3 BsAbs were analyzed in OVCAR-3 cell lines at a concentration of 5 μg/mL. The EpCAM and CLDN3 monoclonal antibodies were used as controls

To further reduce the risk of toxicities in non-malignant tissues, we aim to select clones that exhibit significantly reduced binding and endocytic activities in a monovalent format (Fig. S3). The gp120 antibody was used as a nonbinding arm of EpCAM X gp120 or gp120 X CLDN3 BsAbs with either an EpCAM or CLDN3 monovalent arm [37]. To avoid heavy chain mismatch, we used knob-into-hole technology and crossmab technology to prevent the problem of light chain mismatch [38] (Fig. 2A). Then, we used these monovalent format antibodies against EpCAM or CLDN3 to test their binding and internalization activity in OVCAR-3 and NCI-H1781 cancer cells, ultimately obtaining five EpCAM antibodies and three CLDN3 antibodies that met the requirements. We also detected the affinity of these antibodies in bivalent and monovalent format by Surface Plasmon Resonance (SPR) method, and the results showed that they had good affinities in bivalent format, and clones B and D of EpCAM and three CLDN3 clones still had good affinities in monovalent format (Fig. S6). Next, we constructed 15 EpCAM X CLDN3 bsAbs and tested their binding and internalization activity in OVCAR-3 and NCI-H1781 cancer cells to obtain bsAbs that could restore binding and internalization activities (Fig. S2). As shown in Fig. 2B and C, most bsAbs regained binding and internalization activities compared to the parental monoclonal antibodies. More interestingly, three bsAbs (BsAb 1, EpCAM-A X CLDN3-C; BsAb 2, EpCAM-B X CLDN3-C; BsAb 3, EpCAM-D X CLDN3-C) exhibited even better binding and internalization activities, possibly due to synergistic effects from the presence of two arms (Fig. 2B and C). In addition, we also detected the dose-dependent binding activities of these three BsAbs on OVCAR-3 tumor cells. As shown in Figure S7, compared with their parental monoclonal antibodies, the bsAbs showed better binding activities. Therefore, we choose these three bsAbs to build EpCAM X CLDN3 BsADCs.

### EpCAM X CLDN3 BsADCs exhibit significant anti-tumor activity in vitro and in vivo

**Fig. 3:**
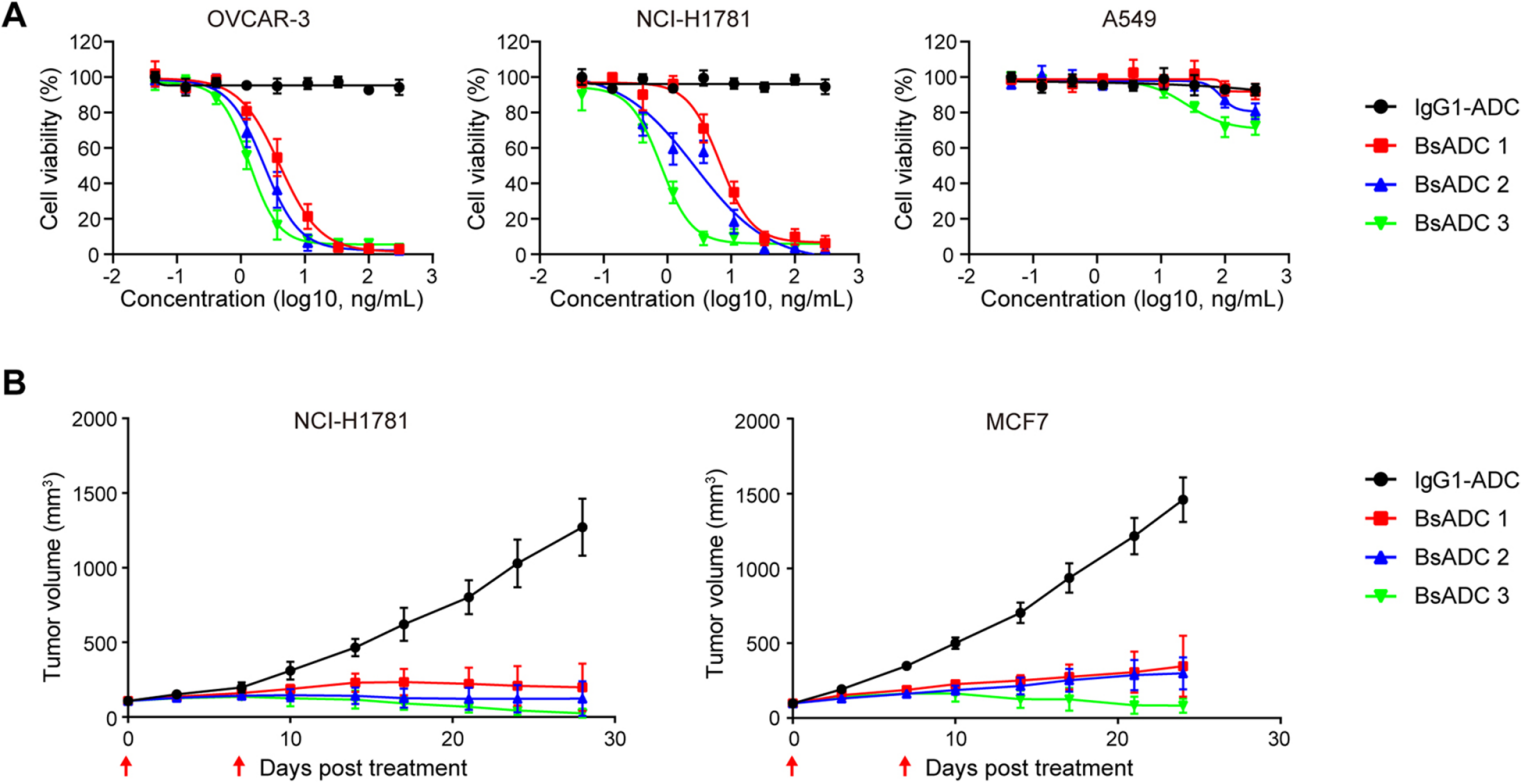
EpCAM X CLDN3 BsADCs demonstrate potent anti-tumor efficacy. **A** In vitro cytotoxicity of EpCAM X CLDN3 BsADCs and control ADC against OVCAR-3, NCI-H1781, and A549 cells. The cancer cells were treated with ADC molecules for 72 h, and cell viability was measured with a CCK-8 kit. **B** The anti-tumor activity of EpCAM X CLDN3 BsADCs in NCI-H1781 and MCF7 xenograft models. Corresponding cancer cells were injected subcutaneously into 7-week-old immunodeficient mice (8 mice per group) at 2 × 10^6^ cells per mouse, and BsADCs (1 mg/kg, twice as indicated by red arrowhead) were intravenously administered when the tumor volume of the mice reached 150 mm^3^. Tumor volumes were measured

Deruxtecan (Dxd) is a potent topoisomerase I inhibitor, and ADCs using Dxd as a payload have demonstrated excellent anti-tumor activity in multiple tumor models [39]. The EpCAM X CLDN3 BsADCs were generated by bio-conjugating the Dxd payload to endogenous cysteine residues on three BsAbs via a GGFG linker. We further characterized the Drug-to-Antibody Ratio (DAR) of these BsADCs using the Hydrophobic Interaction Chromatography (HIC), and the results showed that the DAR of the three BSADCs were 3.9, 4.12, and 4.03, respectively (Fig. S8). The cytotoxic efficacy of BsADCs was examined in a series of in vitro assays utilizing various human cancer cell lines. A dose-dependent in vitro anti-proliferative response to BsADCs was observed in the EpCAM and CLDN3 high-expressing OVCAR-3 and NCI-H1781 cancer cells (Fig. 3A). However, A549 cells with low expression of EpCAM and CLDN3 had a significantly weaker response to BsADCs (Fig. 3A). In contrast, the non-binding control ADC (IgG-ADC) containing the identical linker and payload did not exhibit cell growth inhibitory activities in all three cancer cell lines (Fig. 3A). These results indicate that the cell growth inhibitory activity of BsADCs was remarkably enhanced by drug conjugation to the BsAbs and also that Dxd shows target-specific growth inhibition against EpCAM and CLDN3 positive cell lines.

The in vivo anti-tumor activity of BsADCs was evaluated in mice xenograft models. Mice transplanted with NCI-H1781 or MCF7 cancer cell lines were injected intravenously with BsADCs (1 mg/kg) or control IgG-ADC (1 mg/kg) twice on day 0 and day 7, respectively. A comparison of tumor growth at day 28 showed that BsADCs treatment resulted in significant tumor regression compared to IgG-ADC treatment (Fig. 3B). Furthermore, the BsADC regressions were sustained through an additional extended observation period despite the mice receiving only two doses of the ADC. Consistent with the results of in vitro cancer cell toxicity assays, BsADC 3 showed the best anti-tumor effect in mouse xenograft models (Fig. 3B).

### BsADC 3 exhibits lower toxicity towards normal tissues with EpCAM expression

**Fig. 4:**
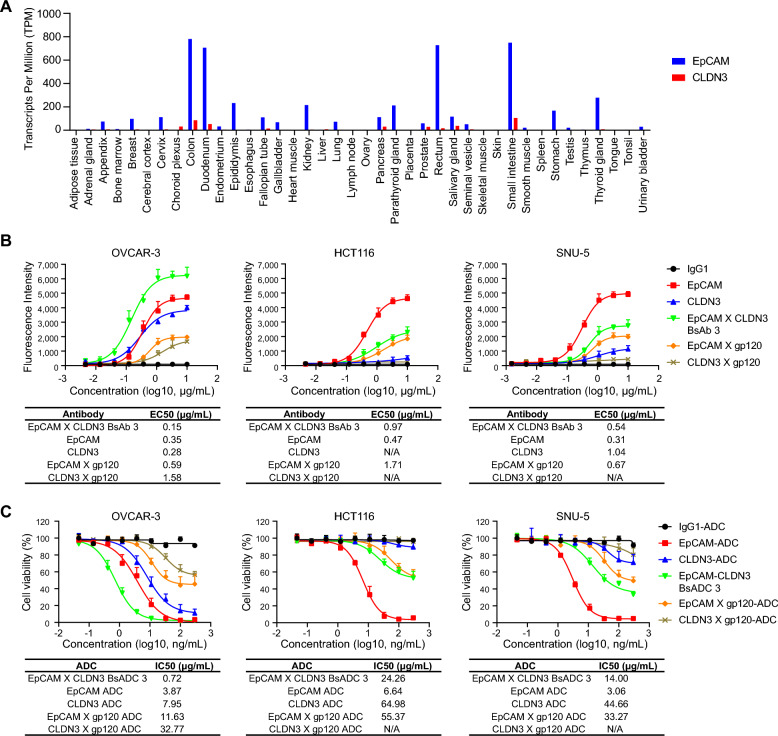
EpCAM X CLDN3 BsADC shows reduced binding and cytotoxic activities on EpCAM^high^ CLDN3^low^ cells. **A** The RNA transcriptional expression of EpCAM and CLDN3 in normal tissues, with data sourced from the Human Protein Atlas database. **B** Compared with EpCAM monoclonal antibody, EpCAM X CLDN3 BsAb increased the binding activity against OVCAR-3 cells with high expression of EpCAM and CLDN3, but decreased the binding activity against HCT116 and SNU-5 cells with high expression of EpCAM and low expression of CLDN3, and the EC50 values were also shown. **C** Compared with EpCAM monoclonal ADC, EpCAM X CLDN3 BsADC increased the tumor growth inhibitory activity against OVCAR-3 cells, but decreased the tumor growth inhibitory activity against HCT116 and SNU-5 cells, and the IC50 values were also shown

EpCAM is moderately expressed in several normal tissues, predominantly in the gastrointestinal tract, kidneys, and pancreas, as indicated by the RNA sequencing expression data from the human protein altas database (Fig. 4A). However, the expression level of CLDN3 in these normal tissues is much lower (Fig. 4A). Because we selected the 1:1 format and deliberately chose clones that would have reduced binding and internalization activities after becoming monovalent structure to generate BsADC, the BsADC 3 molecular should exhibit lower binding and cytotoxic activities towards cells that moderately express EpCAM but have low CLDN3 expression.

To validate this hypothesis, we compared the cell-binding activity of BsAb 3, its parental monoclonal antibodies, and its monovalent antibodies in HCT116 and SNU-5 cells, which highly express EpCAM but have low CLDN3 expression. As shown in Fig. 4B, the binding activity of BsAb 3 to these two cells is very close to that of EpCAM monovalent antibody and much lower than that of EpCAM bivalent parental antibody. Similarly, when we compared the cytotoxic activity of BsADC 3 with its parental bivalent ADCs and its monovalent ADCs in HCT116 and SNU-5 cells, BsADC 3 also showed similar cytotoxic activity to monovalent EpCAM X gp120-ADC (IC50, 24.26 vs 64.98 μg/mL in HCT116, 14.00 vs 44.66 μg/mL in SNU-5) and much lower than that of parental bivalent ADC molecule EpCAM-ADC (IC50, 24.26 vs 6.64 μg/mL in HCT116, 14.00 vs 3.06 μg/mL in SNU-5) (Fig. 4C). Both the CLDN3 parental bivalent ADC and monovalent CLDN3 X gp120-ADC have relative weak cytotoxic activities on HCT116 and SNU-5 cells, which is mainly due to its low expression on these two cells. However, the binding activity of BsAB 3 to OVCAR-3 cells with high expression of both EpCAM and CLDN3 was much higher than that of its two parental bivalent antibodies (Fig. 4B), and the cytotoxic activity of BsADC 3 was also more potent than that of the EpCAM and CLDN3 parental ADCs (IC50, 0.72 vs 3.87 and 7.95 μg/mL) (Fig. 4C).

**Fig. 5:**
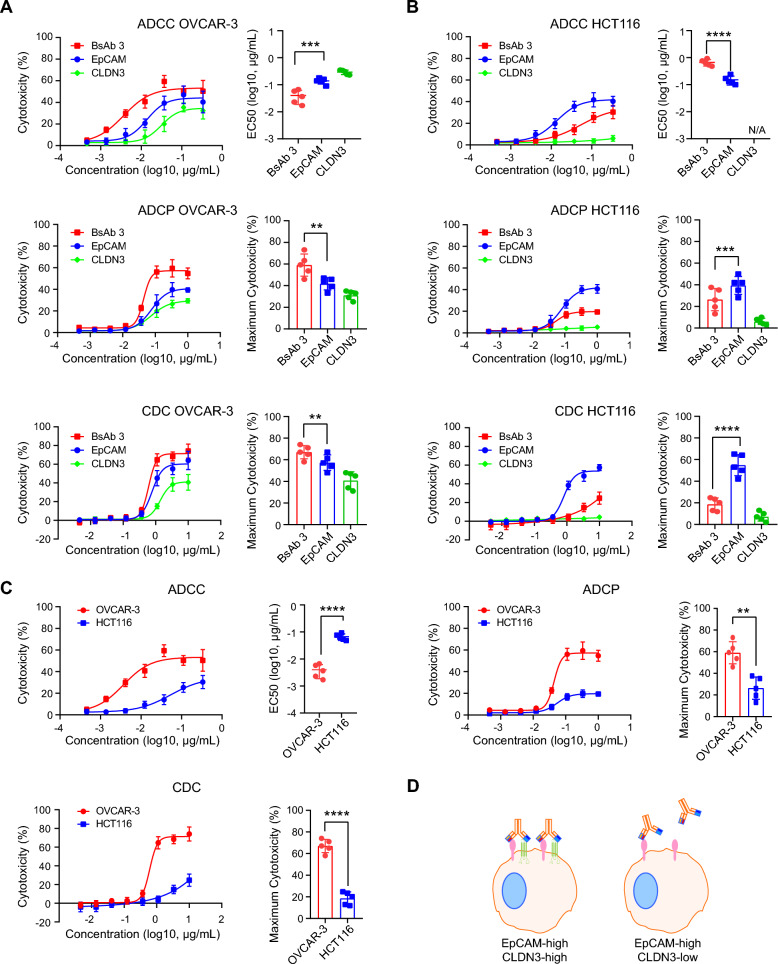
EpCAM X CLDN3 BsAb demonstrates differential ADCC, ADCP, and CDC effects on OVCAR-3 and HCT116 cells. **A** Representative curves of ADCC, ADCP and CDC effects on OVCAR-3 cells. EpCAM X CLDN3 BsAb 3 exhibits a stronger ADCC, ADCP and CDC effects than their parental bivalent antibodies on OVCAR-3 cells, **B** Representative curves of ADCC, ADCP and CDC effects on HCT116-3 cells. EpCAM X CLDN3 BsAb 3 exhibits a weaker ADCC, ADCP and CDC effects than EpCAM parental bivalent antibody on HCT116 cells. CLDN3 parental bivalent antibody has very weak ADCC, ADCP and CDC effects on HCT116 cells, which is mainly due to its extremely low expression on HCT116 cells. **C** Representative curves of ADCC, ADCP, and CDC effects on OVCAR-3 and HCT116 cells. EpCAM X CLDN3 BsAb exhibits a stronger ADCC, ADCP, and CDC effects on OVCAR-3 cells than HCT116 cells. **D** Schematic illustration of the difference between EpCAM X CLDN3 BsAb binding to EpCAM^high^ CLDN3^high^ cells and EpCAM^high^ CLDN3^low^ cells. Data are presented as mean values ± SD. **, *p* < 0.01; ***, *p* < 0.001; ****, *p* < 0.0001

ADC molecules typically use the wild-type IgG1 structure to enhance the anti-tumor activity through the ADCC (Antibody-Dependent Cellular Cytotoxicity), ADCP (Antibody-Dependent Cellular Phagocytosis), and CDC (Complement-Dependent Cytotoxicity) effects of antibodies. Our BsADC molecules have strong binding activities to tumor cells with high EpCAM and CLDN3 expression, and also exhibited stronger ADCC, ADCP, and CDC effects than their parental bivalent antibodies on OVCAR-3 cells (Fig. 5A). However, the ADCC, ADCP, and CDC effects of antibodies can sometimes cause on-target off-tumor toxicity effects on normal tissues. Our BsADC molecules bind weakly to HCT116 cells with high EpCAM expression but low CLDN3 expression, and also exhibited weaker ADCC, ADCP, and CDC effects than the EpCAM parental bivalent antibodies on HCT116 cells (Fig. 5B), thereby might be able to minimize the toxic side effects on normal tissues. Then, we compared the ADCC, ADCP, and CDC effects of the BsAb 3 molecule on OVCAR-3 and HCT116 cells. As shown in Fig. 5C, BsAb 3 showed high ADCC, ADCP, and CDC activities against OVCAR-3 but relatively low activities against HCT116, further suggesting that BsAb 3 might decrease the on-target off-tumor toxicity effects on normal tissues while retaining the cytotoxic activity to tumor cells.

**Fig. 6:**
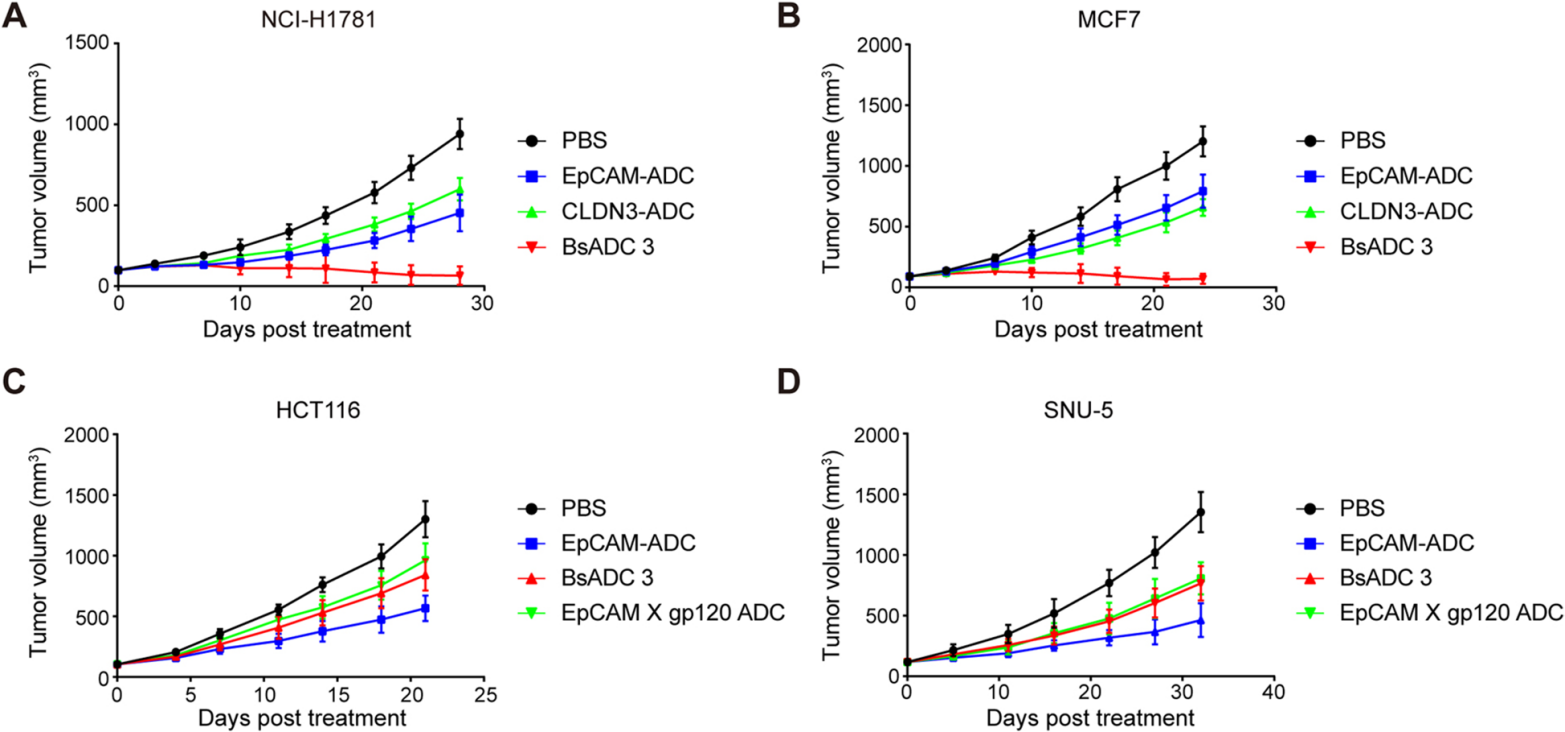
EpCAM X CLDN3 BsADC shows different efficacy on EpCAM^high^ CLDN3^high^ cells and EpCAM^high^ CLDN3^low^ cells in vivo. **A** and **B** CB17/SCID mice (N = 5) engrafted with NCI-H1781 cells (**A**) or MCF7 cells (**B**) (EpCAM^high^CLDN3^high^) randomized on day 12 and treated intravenously immediately with EpCAM X CLDN3 BsADC (1 mg/kg), EpCAM ADC (1 mg/kg), and CLDN3 ADC (1 mg/kg). **C** and **D** CB17/SCID mice (N = 5) engrafted with HCT116 cells (**C**) or SNU-5 cells (**D**) (EpCAM^high^CLDN3^low^) randomized on day 12 and treated intravenously immediately with EpCAM X CLDN3 BsADC (1 mg/kg), EpCAM ADC (1 mg/kg), and EpCAM-gp120 ADC (1 mg/kg). Data are presented as mean values ± SD

Finally, we further validated our hypothesis in mouse models. In the NCI-H1781 and MCF7 models with high expression of both EpCAM and CLDN3, BsADC 3 demonstrated more potent anti-tumor activity than the EpCAM parental monoclonal ADC (Fig. 6A and B). However, in the HCT116 and SNU-5 models with high expression of EpCAM but low expression of CLDN3, the anti-tumor activity of BsADC 3 was close to EpCAM monovalent ADC, significantly lower than that of the EpCAM parental monoclonal ADC (Fig. 6C and D). These results indicate that BsADC 3 can maintain high cytotoxic activity against tumor cells with dual high expression while reducing the cytotoxic activity against cells with moderate EpCAM expression, thereby helping to expand the therapeutic window in the clinic.

### BsADC 3 has good PK and safety profiles in mice model

**Fig. 7:**
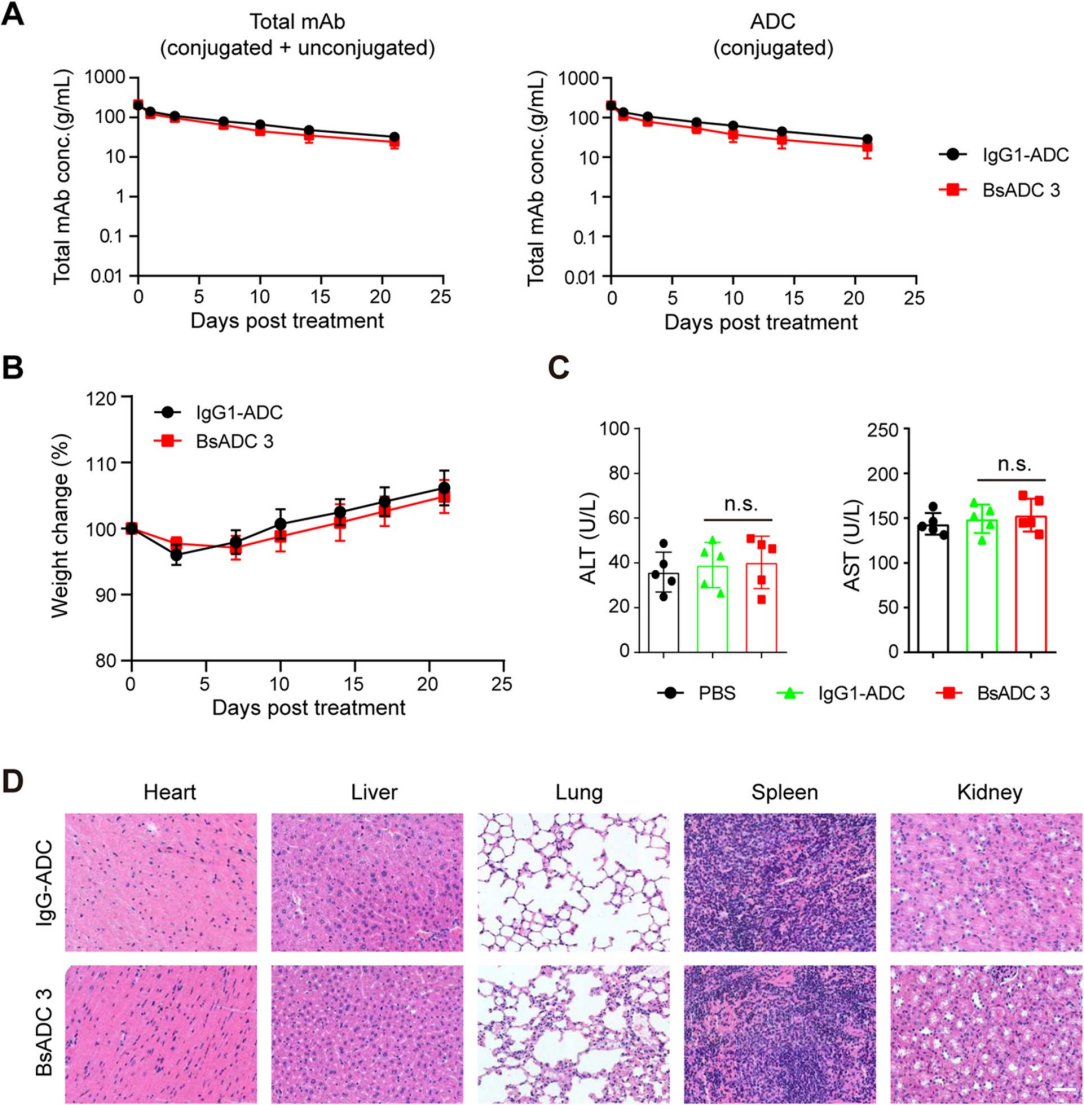
In vivo pharmacokinetics and safety of EpCAM X CLDN3 BsADC. **A** Pharmacokinetics of EpCAM X CLDN3 BsADC in female BALB/c mice. At the indicated time points, blood was collected to quantify total antibody (conjugated and unconjugated, left) and ADC (conjugated only, right) by sandwich ELISA (N = 5). **B** Effect of two-doses of IgG-ADC or BsADC (6 mg/kg) on body weight in female BALB/c mice. **C** The blood chemistry parameters (ALT, left, AST, right) 3 days post injection of three doses of vehicle control or EpCAM X CLDN3 BsADC (6 mg/kg) in female BALB/c mice. Data are presented as mean values ± SD. **D** Representative images of H&E stained the heart, liver, spleen, lung, and kidney tissues of the mice. (Scale bar: 100 μm)

Pharmacokinetic profiles of EpCAM X CLDN3 BsADC 3 and total antibody in non-tumor bearing mice were evaluated after single intravenous administration of BsADC 3 and the IgG-ADC at doses of 10 mg/kg (Fig. 7A). Sandwich ELISA was performed to determine concentrations of total mAb (both conjugated and unconjugated) and intact ADC (conjugated only) in the blood. The half-life for total and conjugated antibodies was 9.2 and 9.7 days, indicating typical IgG1 kinetics and excellent stability in vivo (Fig. 7A). This observation further indicates these antibodies' excellent stability within an in vivo environment, thereby highlighting their potential for therapeutic applications. Furthermore, no significant differences were observed between plasma concentration–time profiles for BsADC and the total antibody, suggesting that the peptide-linker component of BsADC 3 maintains its stability within plasma conditions.

We further characterized the nonclinical safety profile of the BsADC 3 in mice in repeat-dose studies with doses up to 6 mg/kg. Throughout this study, no significant toxicity was observed in either treatment group as determined by monitoring factors such as body weight loss and daily behavioral observations (Fig. 7B). To further assess potential liver toxicity, blood chemistry tests were carried out three days post-final injection of BsADC 3. We quantified the enzymes aspartate aminotransferase (AST) and alanine aminotransferase (ALT), which indicate liver function, and found that these enzymes remained within the normal range (Fig. 7C). These results suggest that the risk of BsADC 3 inducing hepatotoxicity is almost negligible. Similarly, there was no indication of target-dependent toxicity in any of the normal tissues examined, including the heart, liver, spleen, lungs, and kidneys (Fig. 7D). These results indicate that EpCAM X CLDN3 BsADC 3 has good PK and safety profiles and has potential application in the treatment of EpCAM and CLDN3-expressed tumors.

## Discussion

ADCs have emerged as a leading drug class for cancer therapy and are becoming increasingly of interest for therapeutic applications. In contrast to monoclonal ADCs, BsADCs target two TAAs that may not individually be tumor-specific. Theoretically, this approach enhances the selectivity of BsADCs for tumor cells, potentially reducing side effects on healthy tissues. In addition to improving tumor selectivity, BsAb promotes endocytosis by simultaneously binding to two TAAs on tumor cells, thereby improving payload delivery. In the absence of tumor-specific mAb targets or because tumor-selective targets do not always internalize well, BsAbs may offer a better choice than monospecific antibody-based ADCs for tumor-selective delivery of highly potent chemical payloads. In addition, some BsAbs targeting two TAAs that have already been successful in clinical trials could also be further modified to bsADCs, potentially improving the effectiveness of cancer treatments. Amivantamab is a humanized EGFR-cMET BsAb that has shown a managed safety profile and broad-spectrum anti-tumor efficacy in patients with EGFR exon 20 insertion, EGFR C797S mutation, MET amplification, or resistance to third-generation EGFR TKI Osimertinib [40]. AZD9592 is an EGFR-cMET-targeted BsADC conjugated with topoisomerase 1 inhibitor (TOP1i). Preclinical results show that when AZD9592 binds to and is internalized by tumor cells, TOP1i can be released to kill tumor cells more effectively [41].

BsADCs have great potential as one of the most effective biological therapeutics. However, to construct a good BsADC, many critical factors must be considered, such as target selection, format, binding affinity/avidity, and functional activity. Rational target selection fundamentally determines the mechanism of action (MOA) of the BsADC and is the most crucial step for its success. For example, cadherin 17 (CDH17) and guanylate cyclase 2C (GUCY2C) are highly expressed in colorectal cancer but have very low expression in normal tissues [42, 43]. The BsADC targeting CDH17 and GUCY2C can increase the binding and endocytosis activities, thereby enhancing the anti-tumor activities without worrying too much about toxicity issues. A 2:2 structure BsADC targeting CDH17 and GUCY2C using high-affinity bivalent arms can be chosen in this case.

BsADCs constructed with different combinations of targets tend to adopt different structures and select different affinities due to their distinct MOAs. We take EGFR, an extremely attractive TAA target, as an example. Since the bivalent binding of cMET typically leads to activation rather than inhibition due to its dimerization properties [44], Amivantamab and AZD9592 employ a 1:1 format [37, 41]. In contrast, BL-B01D1, a novel BsADC targeting EGFR and HER3, has shown promising anti-tumor efficacy in clinical trials and utilizes the 2:2 format [45]. Unlike cMET, dimerization of HER3 can neither activate downstream signaling nor stimulate tumor growth due to the absence of an intracellular signal transduction domain, so a bivalent structure with more vital binding ability can be adopted. However, both AZD9592 and BL-B01D1 use a TAA target with a more specific expression in combination with EGFR. This approach increases therapeutic efficacy, but the patients who can benefit must have high expression of both TAA targets, thus narrowing the range of patients who can benefit. Another strategy is to select a TAA that is highly expressed in various tumors in combination with EGFR, such as MUC1 and B7H3 [46, 47]. In most patients with high EGFR expression, MUC1 or B7H3 are also highly expressed, so theoretically, all patients with high EGFR expression could benefit from these BsADCs. Since the expression specificity of MUC1 or B7H3 is not very high, the 1:1 format is usually used, and the affinity of MUC1 or B7H3 is not expected to be too high.

Compared with BsAbs, which target two TAA targets, BsADCs have a more comprehensive range of target selection. Generally, the two TAA targets of BsAbs usually mediate signal transduction and promote tumor growth. Besides killing tumor cells through ADCC activity or promoting target degradation via endocytosis, BsAbs often need high affinity to block ligand binding and thus block signal transduction. For example, the EGFR and cMET arms of Amivantamab have high affinity and can block signal transduction. If one of them does not mediate signal transduction, it is likely to be less effective, such as EGFR-HER3. Duligotuzumab (MEHD7945A), a BsAb targeting EGFR and HER3, showed no clinical benefit in comparison to cetuximab (anti-EGFR mAb) in phase 2 trials in patients with metastatic colorectal cancer or head and neck squamous cell carcinoma [48]. Expression of HER3 determined by RNA or protein in tumor biopsies did not correlate with the response rate to duligotuzumab. Therefore, the researchers concluded HER3 has a minor role in EGFR inhibitor naïve mCRC patients [48]. However, BL-B01D1, a BsADC targeting EGFR and HER3, has shown promising anti-tumor efficacy in clinical trials [49]. These findings suggest that more TAA targets, especially those that do not mediate signal transduction, can be used to design combinations for unmet clinical needs through BsADCs.

Although CLDN18.2 and CLDN6 are recognized as promising tumor-associated antigen (TAA) genes, CLDN6 is highly expressed in ovary and testicular germ cell tumors, and CLDN18.2 is highly expressed in pancreatic and stomach tumors (Fig. S9). The correlation analysis results also indicate that EpCAM and CLDN6 are only co-expressed in ovary tumor, but not in other tumors, such as stomach and Colorectal tumors. EpCAM and CLDN18.2 are only co-expressed in stomach tumor, but not in other tumors (Fig. S9). CLDN3 and EPCAM are both highly expressed in many tumors, so they are more suitable to generate BsADCs. Meanwhile, CLDN3 has much lower expression in normal tissues, making it an attractive target for developing antibody-based therapeutics. There is a high degree of sequence homology between CLDN3 and other family members; for instance, CLDN4, CLDN6, CLDN8, and CLDN9 differ from it by only a few amino acids in their extracellular domains [50]. The discovery of a particular antibody that selectively binds to CLDN3 without interacting with other family members is crucial for unintentionally amplifying the off-target effects of drug toxicity. In 2014, Eisai Co., Ltd developed an antibody with a high affinity for both CLDN3 and CLDN4 [51]. Although it has shown impressive anti-tumor activity in preclinical mouse models, its potential clinical use may face obstacles due to CLDN4's widespread expression in many normal tissues, which raises concerns about possible toxicity in healthy tissues. Here, we generated a CLDN3-specific antibody without binding to CLDN4, CLDN6, CLDN8, and CLDN9. We then used it to construct EpCAM X CLDN3 BsADC and further demonstrated that it has good efficacy in treating a variety of tumors in vitro and in vivo. Its specificity provides a significant advantage in addressing potential toxicity issues during future drug development.

While both monospecific ADCs and BsAbs have been established as potent and credible options for cancer therapy, BsADCs are still in the exploration stage. Still, BsADCs may play a more significant role in the future due to their special MOAs and advantages. BsADCs are engineered to increase the selectivity of payload delivery, enhance its internalization to improve efficacy, minimize the risk of toxicities in non-malignant tissues, and overcome the escape mechanisms of tumor cells. We are confident that BsADC-based therapies could revolutionize existing cancer treatment options and represent a significant forward in combating cancer.

## Supplementary Information

Supplementary Material 1.

## Data Availability

No datasets were generated or analysed during the current study.
