# Supplementary material for: Development of a bispecific antibody–drug conjugate targeting EpCAM and CLDN3 for the treatment of multiple solid tumors
Source: Exp Hematol Oncol. 2025 Mar 8;14:33. doi: 10.1186/s40164-025-00624-9 (PMC11889805; doi:10.1186/s40164-025-00624-9)
Supplement: Supplementary file 1 — Supplementary Material 1. [file 40164_2025_624_MOESM1_ESM.docx]

**Supplementary Figures and Table**

**
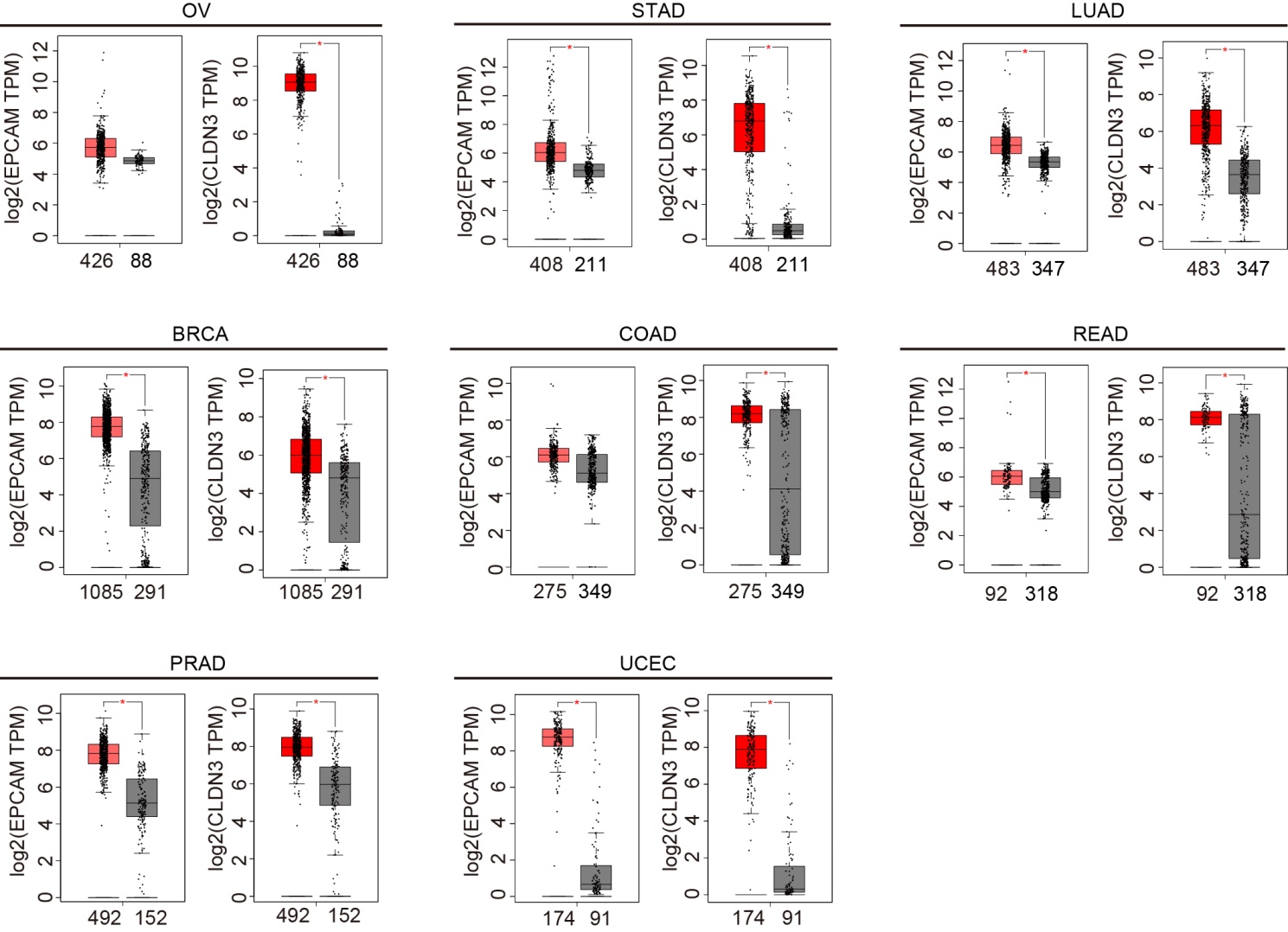
**

**Supplementary Fig. 1. EpCAM and CLDN3 are highly expressed in various types of tumors.**

The GEPIA database shows that EpCAM and CLDN3 are highly expressed in various types of tumors. Tumor samples are represented in red, normal samples in gray, and the corresponding sample numbers are labeled. Abbreviations for different cancers are shown in supplementary table 1.


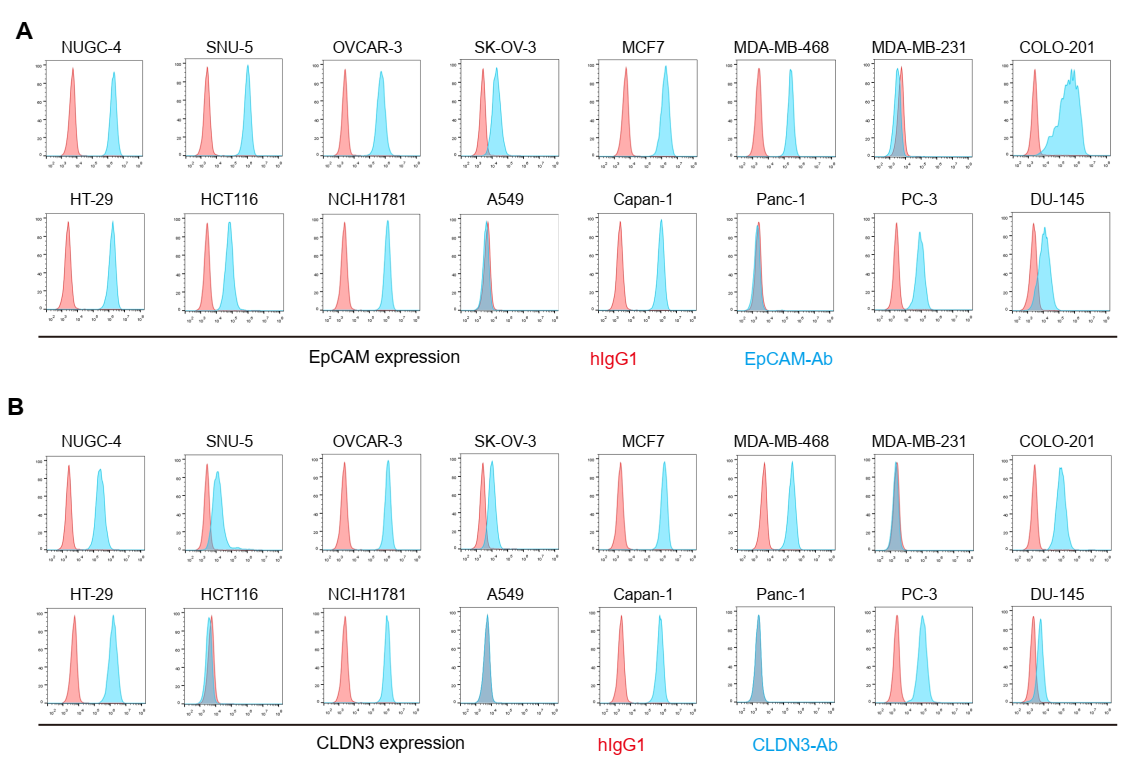


**Supplementary Fig. 2. Expression of EpCAM and CLDN3 in different cancer cell lines**

The expression levels of EpCAM (A) and CLDN3 (B) in different cancer cell lines were detected by FACS assay. The data showed that EpCAM and CLDN3 were highly co-expressed in a variety of tumor cells.

**
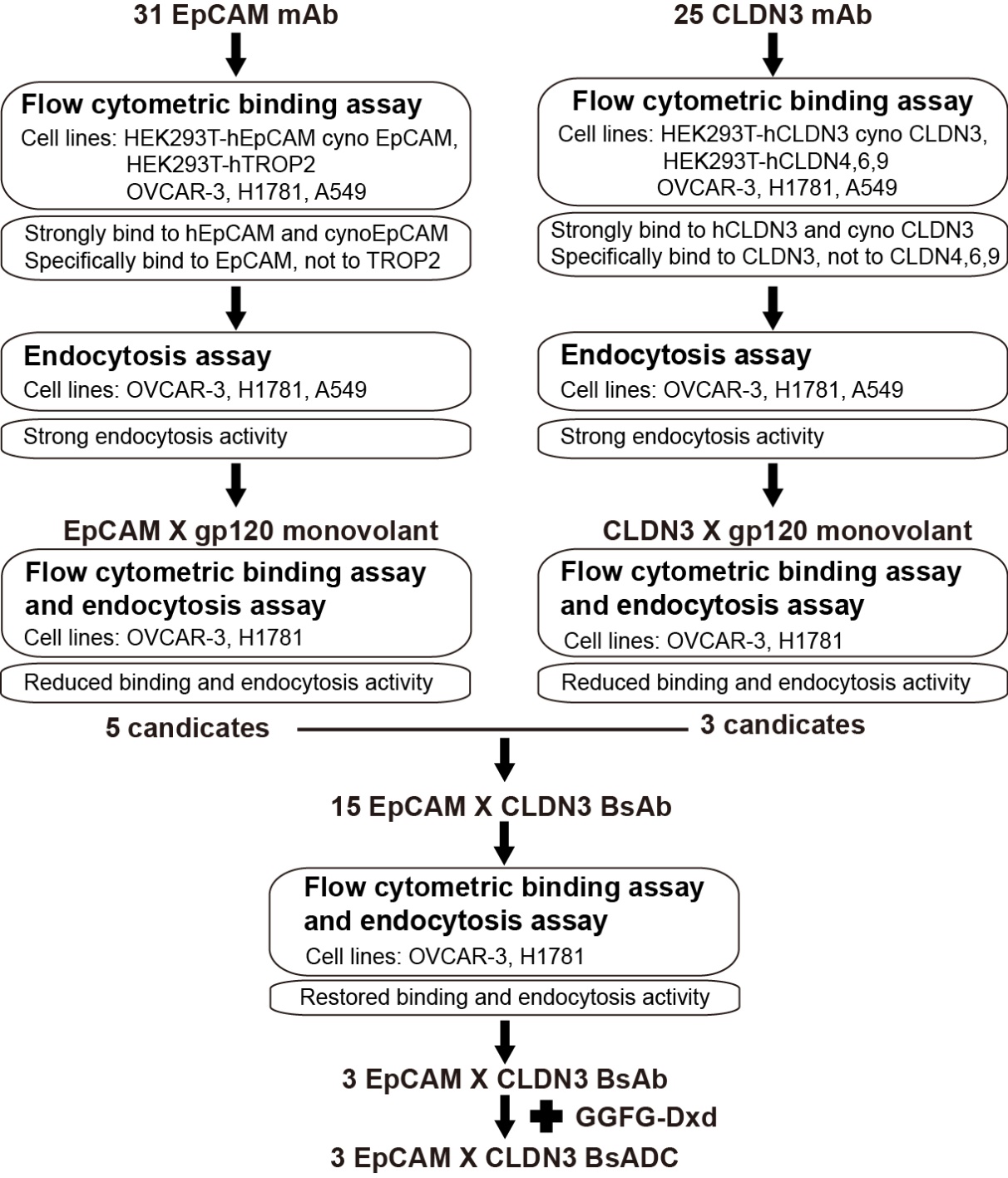
**

**Supplementary Fig. 3. The screening process for monoclonal and bispecific antibodies.**

Screen for monoclonal antibodies with high binding and high endocytic activity, construct them as monovalent antibodies, and select clones with significantly reduced binding and endocytic activity. Then use these clones to construct bispecific antibodies, and select antibodies with restored binding and endocytic activity as candidate bispecific antibodies.

**
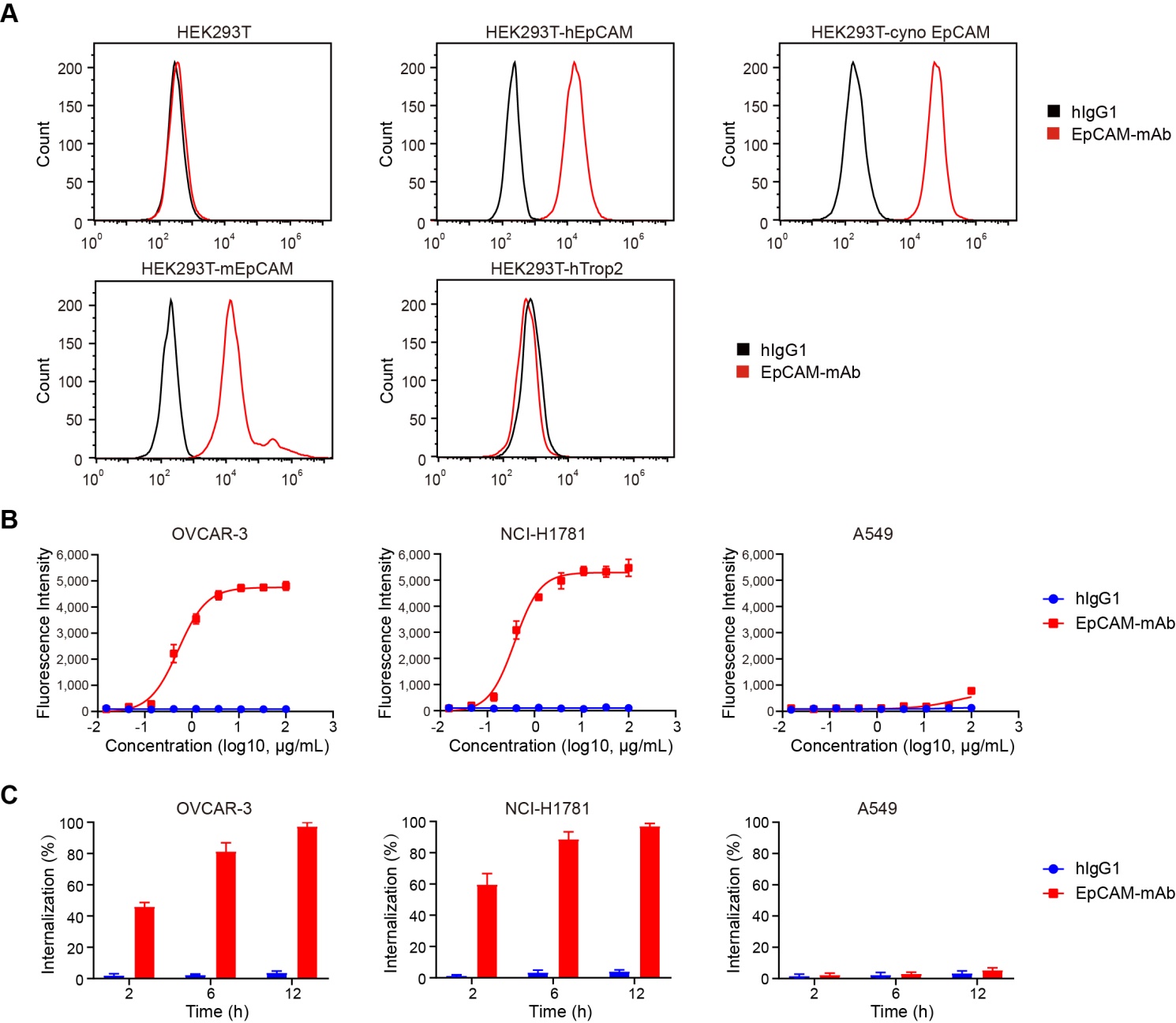
**

**Supplementary Fig. 4. Development and Characterization of EpCAM monoclonal antibodies.**

(A) The representative EpCAM monoclonal antibody binds to HEK293T cells transfected with human, cynomolgus monkey or mouse EpCAM constructs, but not to HEK293T cells transfected with human Trop2 construct.

(B) The representative EpCAM monoclonal antibody strongly binds to OVCAR-3 and NCI-H1781 tumor cells with high EpCAM expression, but weakly binds to A549 tumor cells with low EpCAM expression.

(C) The representative EpCAM monoclonal antibody showed strong internalization activity in OVCAR-3 and NCI-H1781 tumor cells, but weak internalization activity in A549 cells.

**
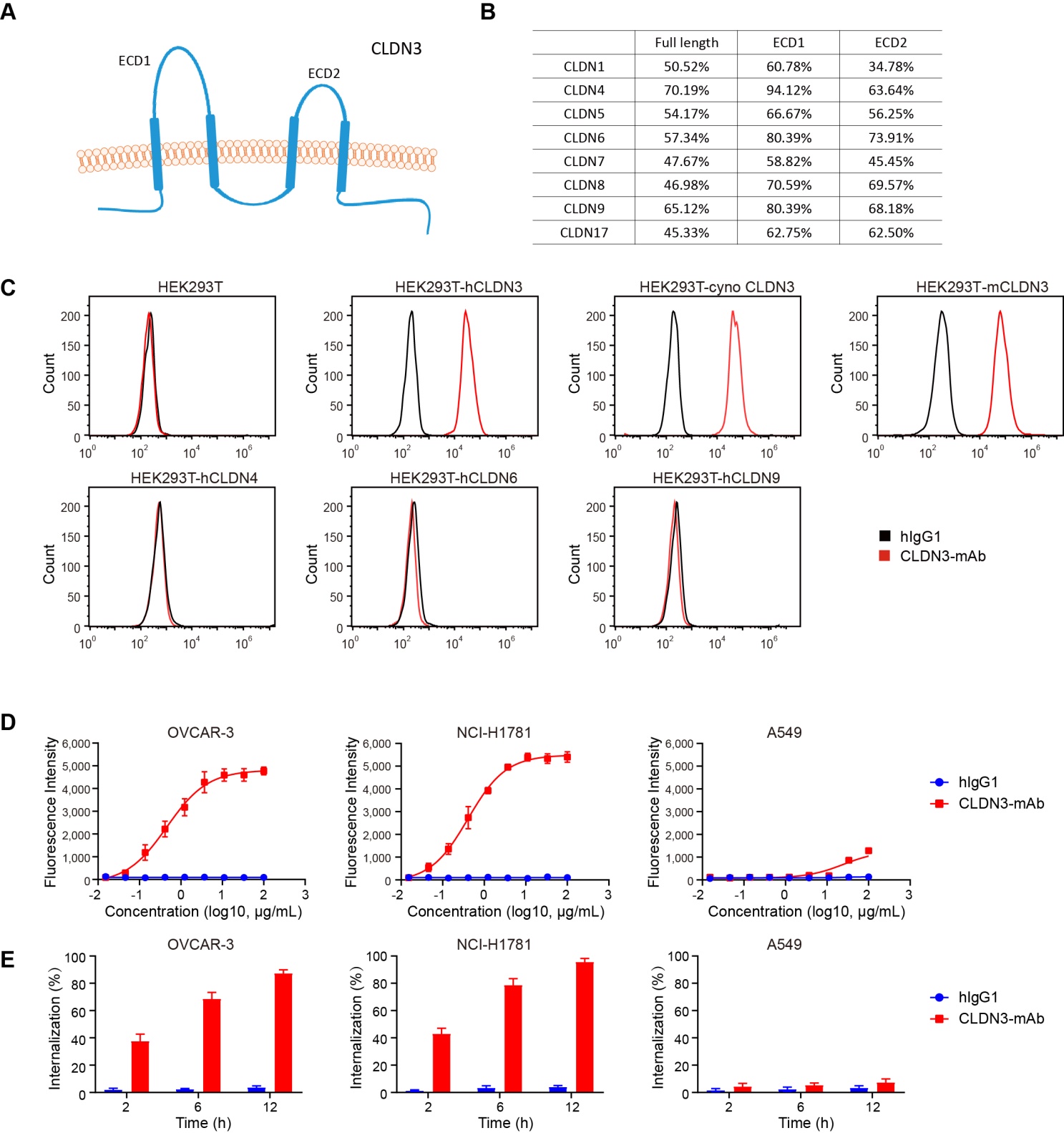
**

**Supplementary Fig. 5. Development and Characterization of CLDN3 monoclonal antibodies.**

(A) Structure diagram of CLDN3 protein. CLDN3 is a four-transmembrane protein with two extracellular domains.

(B) CLDN3 shares a high sequence homology with eight members of the same family. The homology of their full length coding region, extracellular domain 1, and extracellular domain 2 is shown in the table.

(C) The representative CLDN3 monoclonal antibody binds to HEK293T cells transfected with human, cynomolgus monkey or mouse CLDN3 constructs, but not to HEK293T cells transfected with human CLDN4, CLDN6, or CLDN9 constructs.

(D) The representative CLDN3 monoclonal antibody strongly binds to OVCAR-3 and NCI-H1781 tumor cells with high CLDN3 expression, but weakly binds to A549 tumor cells with low CLDN3 expression.

(E) The representative CLDN3 monoclonal antibody showed strong internalization activity in OVCAR-3 and NCI-H1781 tumor cells, but weak internalization activity in A549 cells.

**
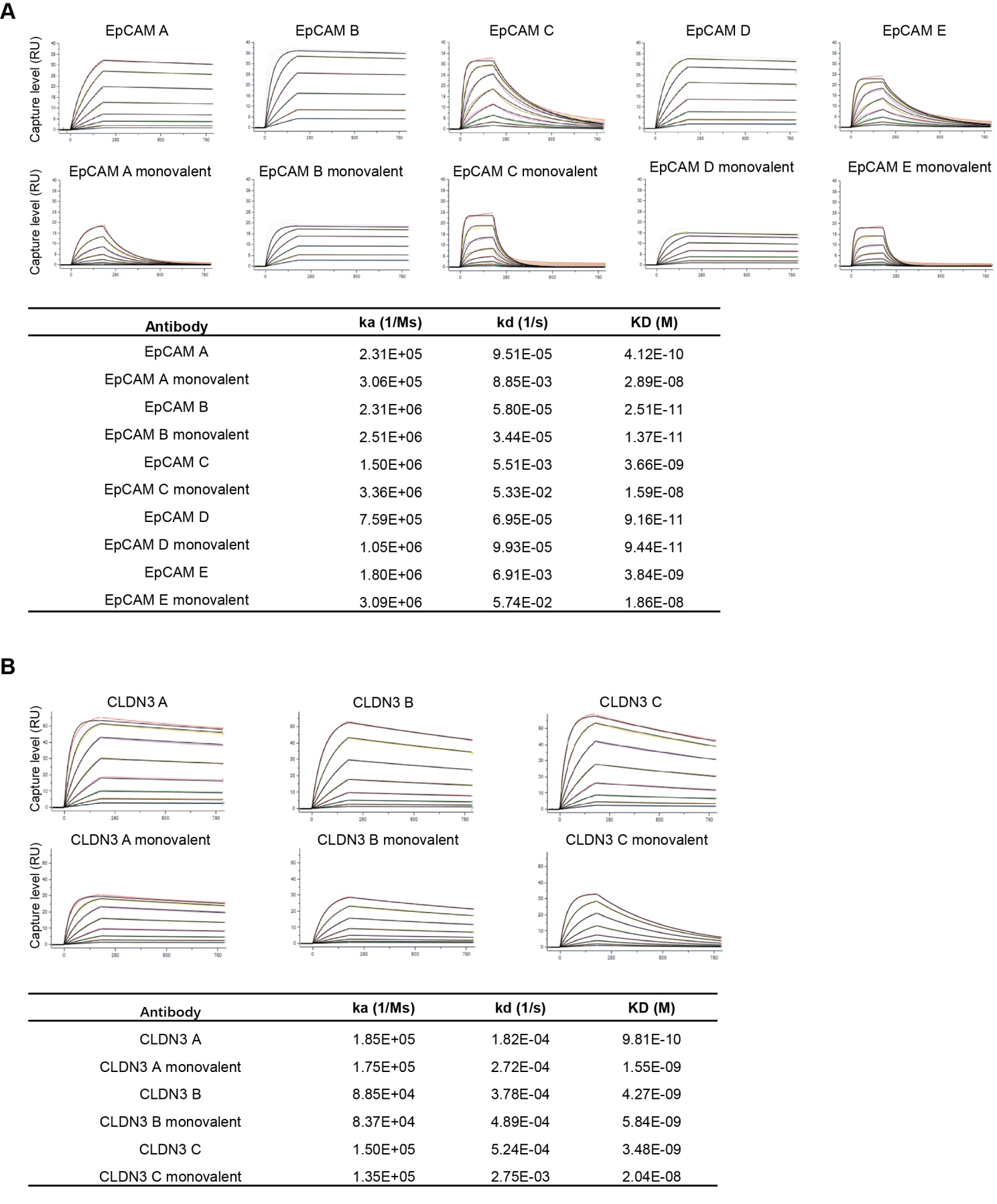
**

**Supplementary Fig. 6. The affinity of monoclonal and monovalent antibodies.**

The affinity of monoclonal and monovalent antibodies of EpCAM (A) and CLDN3 (B) was detected by SPR. The Ka, Kd, and KD values were also shown.

**
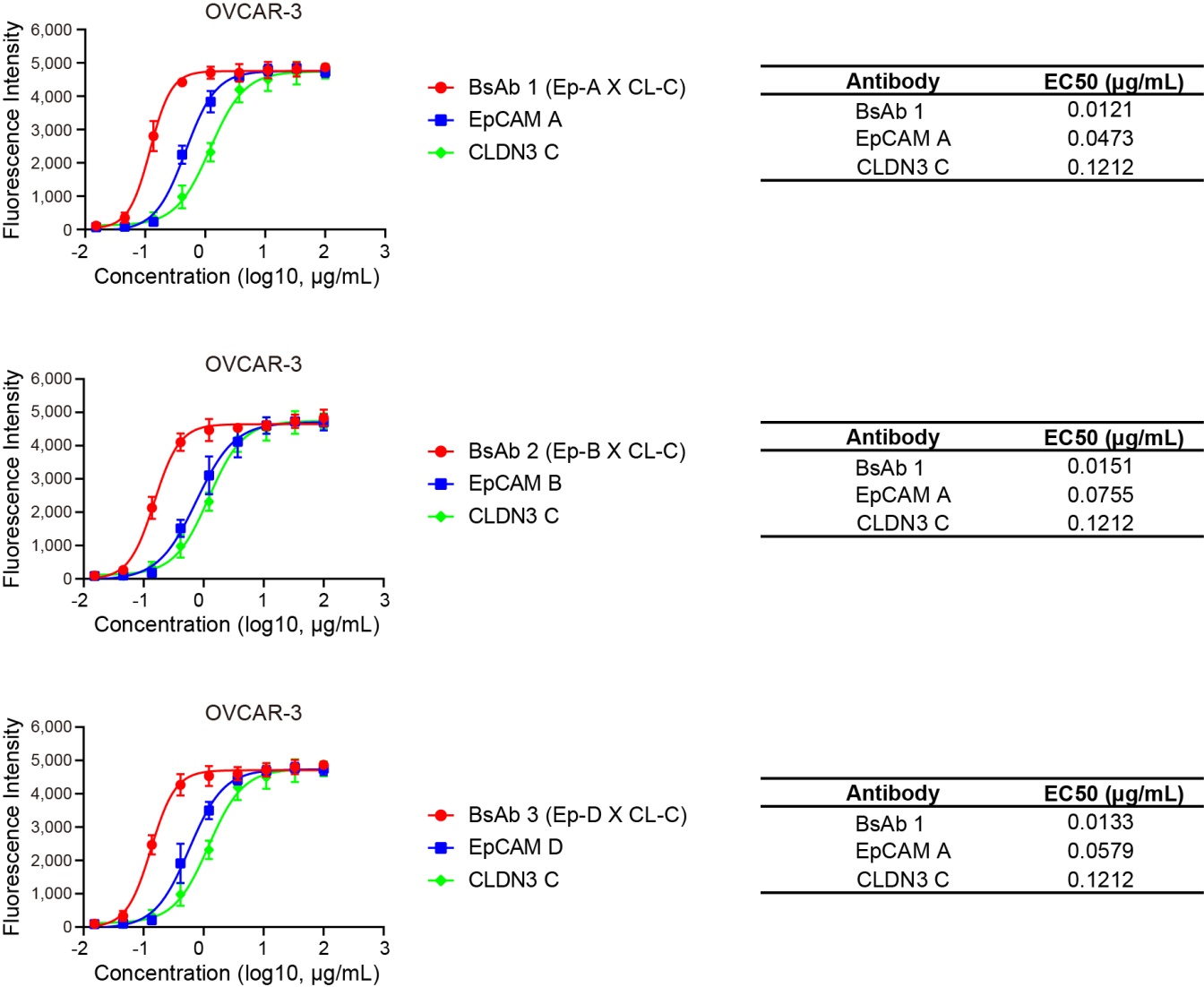
**

**Supplementary Fig. 7. The BsAbs have better binding activities than their parental EpCAM and CLDN3 bivalent antibodies.**

The three EpCAM X CLDN3 BsAs have better binding activities to OVCAR-3 cancer cells, and the EC50 values were also shown.

**
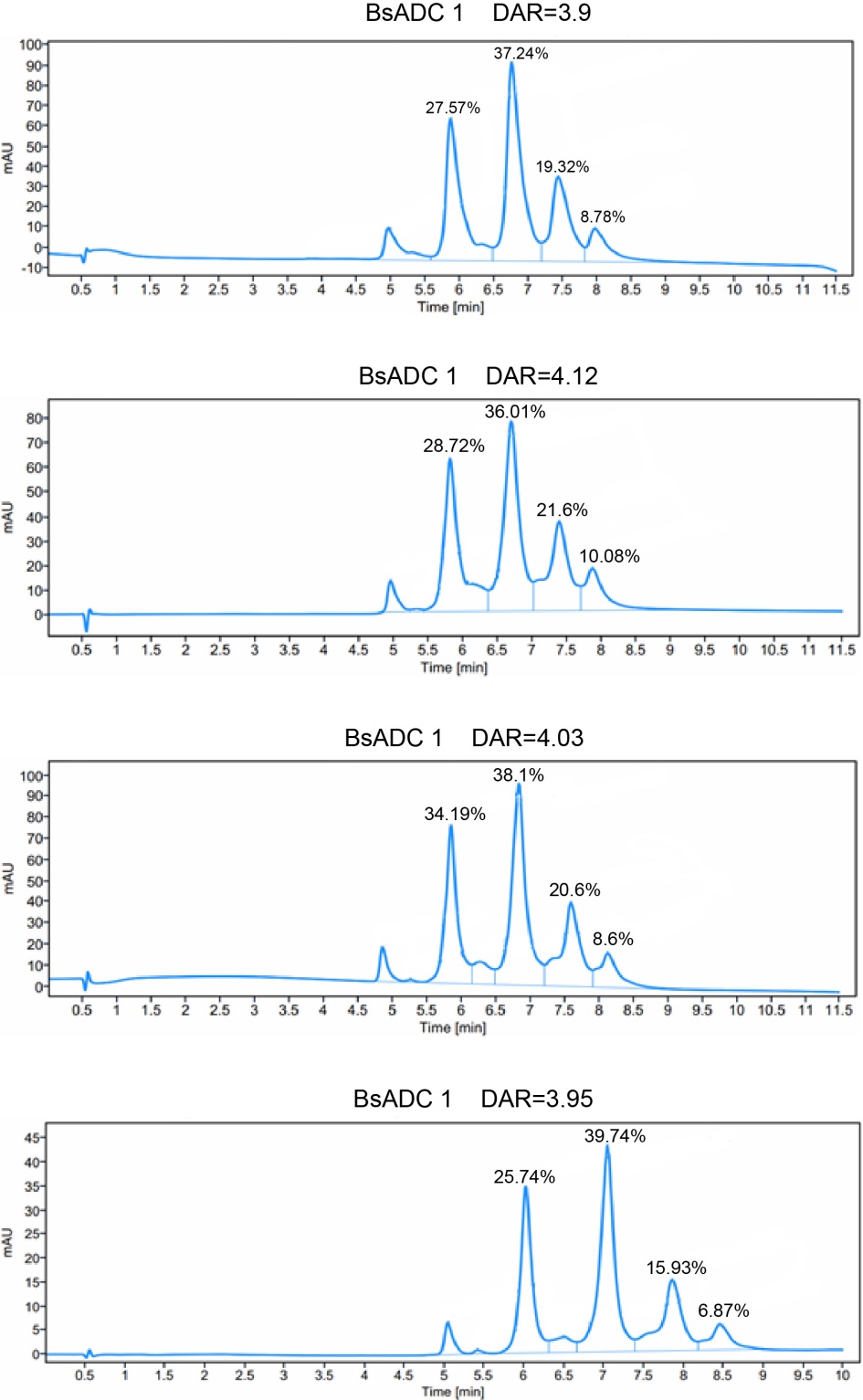
**

**Supplementary Fig. 8. The conjugated drug distribution was detected by HIC-HPLC.**

The HIC-HPLC detection showed that the DAR values of the three BsADCs and IgG1-ADC. The DAR values of BsADCs were 3.9, 4.12 and 4.03, and the DAR of IgG1-ADC was 3.95.

**
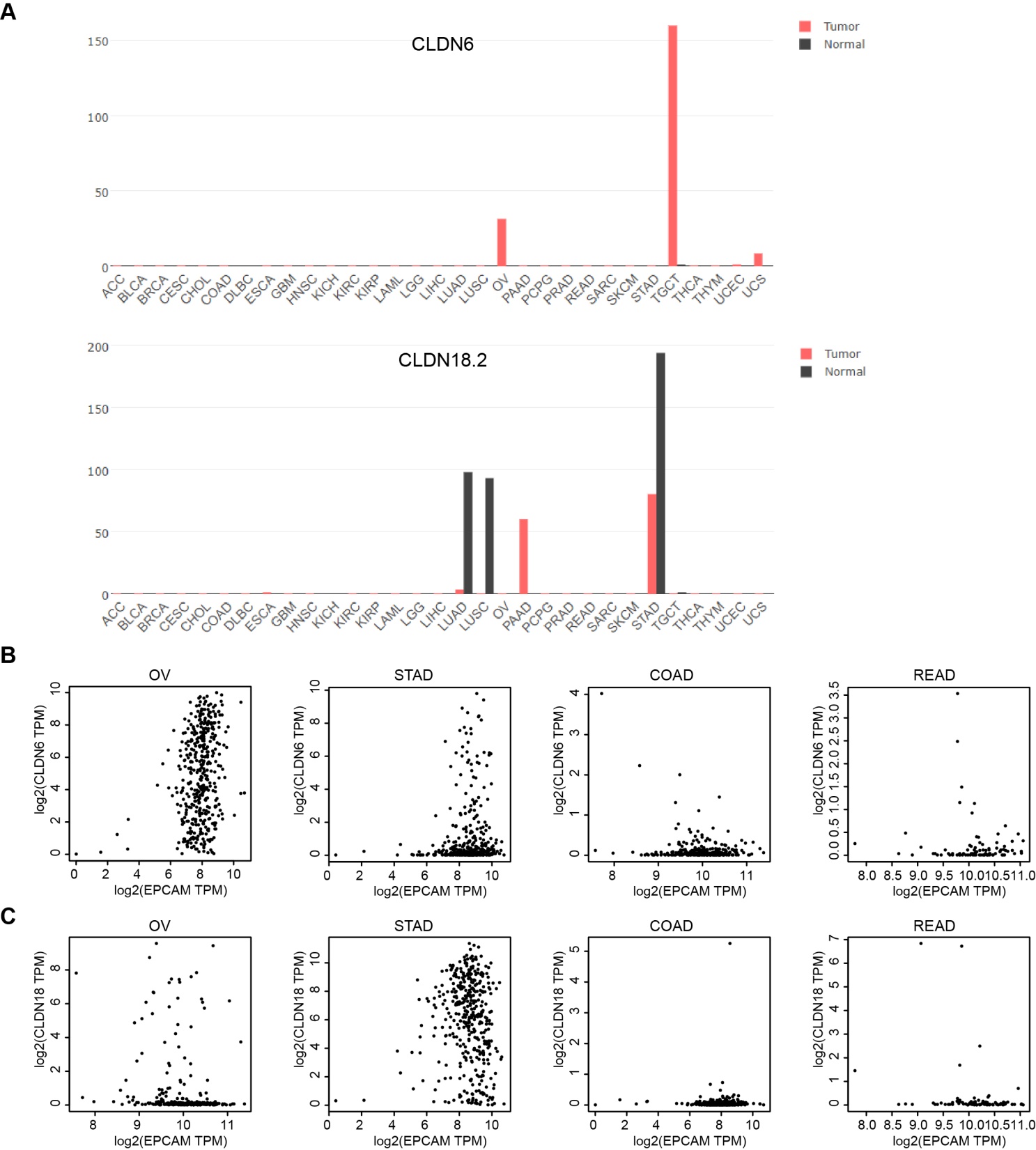
**

**Supplementary Fig. 9. The expression of CLDN6 and CLDN18.2 in tumor cells is not similar to EPCAM.**

(A) Expression of RNA transcription of CLDN6 and CLDN18.2 in the GEPIA cancer tissue sample database. CLDN6 is highly expressed in OV and TGCT tumors, and CLDN18.2 is highly expressed in PAAD and STAD tumors.

(B) The correlation analysis results also indicate that EpCAM and CLDN6 are only co-expressed in OV tumor, but not in STAD, COAD, and READ tumors.

(C) The correlation analysis results also indicate that EpCAM and CLDN18.2 are only co-expressed in STAD tumor, but not in OV, COAD, and READ tumors.

| ACC | Adrenocortical carcinoma |
| --- | --- |
| BLCA | Bladder Urothelial Carcinoma |
| BRCA | Breast invasive carcinoma |
| CESC | Cervical squamous cell carcinoma and endocervical adenocarcinoma |
| CHOL | Cholangio carcinoma |
| COAD | Colon adenocarcinoma |
| DLBC | Lymphoid Neoplasm Diffuse Large B-cell Lymphoma |
| ESCA | Esophageal carcinoma |
| GBM | Glioblastoma multiforme |
| HNSC | Head and Neck squamous cell carcinoma |
| KICH | KICH-Kidney Chromophobe |
| KIRC | Kidney renal clear cell carcinoma |
| KIRP | Kidney renal papillary cell carcinoma |
| LAML | Acute Myeloid Leukemia |
| LGG | Brain Lower Grade Glioma |
| LIHC | Liver hepatocellular carcinoma |
| LUAD | Lung adenocarcinoma |
| LUSC | Lung squamous cell carcinoma |
| MESO | Mesothelioma |
| OV | Ovarian serous cystadenocarcinoma |
| PAAD | Pancreatic adenocarcinoma, |
| PCPG | Pheochromocytoma and Paraganglioma |
| PRAD | Prostate adenocarcinoma |
| READ | Rectum adenocarcinoma |
| SARC | Sarcoma |
| SKCM | Skin Cutaneous Melanoma |
| STAD | Stomach adenocarcinoma |
| TGCT | Testicular Germ Cell Tumors |
| THCA | Thyroid carcinoma |
| THYM | Thymoma |
| UCEC | Uterine Corpus Endometrial Carcinoma |
| UCS | Uterine Carcinosarcoma |
| UVM | Uveal Melanoma |

**Supplementary Table 1. The abbreviations of different cancers.**
